# Agent-based simulation for multi-resource-constrained scheduling of scattered atypical repetitive projects

**DOI:** 10.1038/s41598-026-42832-1

**Published:** 2026-04-03

**Authors:** Rawan Abdelrahman Sultan, Khaled Hamdy, Yasmeen A. S. Essawy

**Affiliations:** 1 Department of Structural Engineering, Ain Shams University (ASU), Cairo, Egypt; 2 Department of Construction Engineering, The American University in Cairo (AUC), Cairo, Egypt

**Keywords:** Agent-based modeling, Stochastic simulation, Spatial scheduling, Geographically dispersed repetitive projects, Crew allocation, Makespan minimization, Engineering, Mathematics and computing

## Abstract

Conventional planning tools struggle to manage the logistical complexity of scattered, short-duration atypical repetitive projects. Although simulation-based planning is well documented, its practical adoption in construction remains limited. This study proposes a simulation–optimization framework implemented in AnyLogic that integrates GIS-enabled agent-based modeling, stochastic simulation, and a GA-based optimization experiment to enable exploration of multi-resource-constrained scheduling decisions. Conceptually informed by multi-mode resource-constrained time–cost trade-off and multiple traveling-salesman formulations, the framework explores makespan-oriented resource-allocation and sequencing configurations under capacity, readiness, and spatial feasibility constraints. Key components include: (i) an adaptive scheduling algorithm for dispersed sites, (ii) a distance-aware rule-based assignment heuristic that supports continuity while limiting avoidable travel, (iii) stochastic disruption modeling, and (iv) spatially explicit visualizations to aid managerial interpretation. Applied to a real-world telecom megaproject, the framework consistently generated substantially shorter schedules across stochastic replications, achieving 46.25% reduction in project duration relative to the practitioner’s baseline heuristic plan. Travel time/share, crew idle time, and utilization were recorded as emergent performance metrics and were used to evaluate operational behavior alongside makespan. Limitations include the absence of real-time traffic data, explicit cost modeling, and distance-penalty functions. Future work will incorporate economic and distance-based constraints and broaden comparative evaluation against alternative scheduling approaches.

**Supplementary Information:**

The online version contains supplementary material available at 10.1038/s41598-026-42832-1.

## Introduction

Simulation has become an essential tool for construction planning, offering means to analyze uncertainty, optimize workflows, and enhance decision-making ^1,2^. Despite decades of documented benefits ^1,3^, its adoption in construction management remains modest ^4–6^, especially compared with manufacturing and logistics ^7,8^. Barriers include the prevalence of highly customized, project-specific models ^9,10^ and the lack of accessible, user-friendly simulation platforms. Consequently, practitioners still rely on heuristic schedules and Excel-based tools, which are poorly suited for the spatial and temporal complexities of modern large-scale projects.

Repetitive construction projects—vertical (e.g., high-rises) ^11^ or horizontal (e.g., roads, pipelines) ^12–14^ may be typical (uniform units) or atypical (heterogeneous conditions) ^15^. A growing subset, Scattered Repetitive Projects (SRPs), involves multiple geographically dispersed units under a single program (e.g., 50 mobile towers, 30 housing units, 20 public buildings). Each unit follows a similar sequence, but unlike linear or vertical arrangements, SRPs lack contiguity, raising logistical challenges ^16,17^. SRPs operate under strict deadlines, limited resources, and crew continuity constraints ^18,19^. Maintaining uninterrupted crew flow can reduce remobilization and idle time, but it is considerably harder to achieve in scattered atypical repetitive projects (SARPs), where site heterogeneity, non-uniform durations, and geographic dispersion make it difficult to preserve constant productivity levels and disrupt sequential crew progression ^13,15,16^. Conventional planning tools offer limited support: Critical Path Method (CPM) fails to capture multi-site routing dependencies ^20–22^, Line of Balance (LOB) and Linear Scheduling Method (LSM) improve visual sequencing ^11,12,20^, but break down under atypical, scattered conditions ^13,15,16^, and commercial tools (e.g., TILOS, VICO) lack dynamic site sequencing and spatially informed routing capabilities.

The urgency of better SARPs scheduling has grown with infrastructure expansion, including roads, utilities, and mobile towers ^23,24^. The global 5G infrastructure market alone is projected to exceed $660 billion by 2030 ^25^, with total infrastructure investments reaching US $94–97 trillion by 2040 ^23^. Mobile tower deployment, in particular, continues to expand into remote, heterogeneous locations ^26,27^. Such environments demand planning frameworks capable of coordinating dispersed crews and compressing makespan under uncertainty, while evaluating travel and idle-time impacts ^16,28^. Prior SRP/SARP scheduling research has advanced optimization formulations, yet there remains a gap in operationally grounded planning for scattered atypical programs where decisions must jointly account for (i) multi-crew deployment and continuity, (ii) spatial routing across dispersed sites, and (iii) stochastic disruptions within a single executable model. Existing methods typically address these elements in isolation or under simplified assumptions, limiting their practical transferability to diverse programs.

This study fills this gap by proposing a simulation-based planning framework for SARPs that integrates Geographic Information System-enabled (GIS-enabled) Agent-Based Modeling (ABM), a Genetic Algorithm-based (GA-based) optimization layer, and stochastic routines to generate feasible contractor-to-site assignments and sequencing under capacity, readiness, and spatial feasibility constraints, and by validating performance on a real-world megaproject and benchmark instances. In this paper, we use “SRPs” to denote the broader class of scattered repetitive projects and “SARPs” to denote the atypical subset that is the primary focus of this study; however, the proposed framework is general and can be applied to both typical and atypical SRPs. Contractors and sites are modeled as autonomous agents interacting within a GIS-enabled environment, allowing spatial routing, task readiness, and capacity constraints to emerge dynamically.

The framework merges principles from the Multi-Mode Resource-Constrained Time–Cost Tradeoff Problem for Repetitive Projects (MRCTCTP-RP) and the Multiple Traveling Salesman Problem (MTSP), thereby capturing both temporal and spatial constraints. Validation on a real-world case study and benchmark problems demonstrates that the ABM–GIS framework can substantially reduce project makespan relative to practitioner-driven heuristics; idle time, travel share, utilization, and continuity patterns are reported as emergent performance metrics to assess operational behavior.

## Literature review

Repetitive construction projects involve performing similar tasks across multiple units under strict time and resource constraints. Optimization efforts typically aim to: (1) reduce duration and cost, (2) minimize idle time, (3) maintain continuity, and (4) improve sequencing and resource efficiency ^18,20,29^. To address these objectives, three main optimization streams have emerged: mathematical programming, heuristic rule-based methods, and metaheuristic algorithms. Table 1 summarizes representative studies in each stream and highlights their objectives, methods, and modeled features (e.g., interruptions, learning effects, soft logic, and multiple crews), showing that while duration/cost optimization is common, support for these operational complexities remains uneven across the literature.

**Table 1:** Previous methods developed to optimize repetitive projects (✓ indicates the feature is explicitly modeled in the referenced study).

Type	Method	References	Optimization objective		Supports interruptions	Learning effect	Soft logic	Multiple number of crews
			Duration	Cost				
Mathematical Methods	Linear Programming	^30^		✓				
		^31^	✓	✓				✓
		^32^	✓	✓	✓			
	Nonlinear Programming	^33^	✓		✓			✓
	Constraint Programming	^34^	✓		✓			
		^15^		✓	✓		✓	
	Dynamic Programming	^21^		✓				
		^35^		✓				
		^36^	✓		✓			
		^29^	✓	✓			✓	✓
	Fuzzy Dynamic Programming	^37^	✓	✓				✓
Heuristic Methods	Permutation Tree-Based	^38^	✓	✓				✓
	LOB-SOM	^39^	✓		✓			✓
Metaheuristic Methods	GA	^40^		✓	✓			✓
		^20^	✓		✓			
		^41^		✓	✓			✓
		^42^	✓	✓			✓	
	Fuzzy GA	^43^	✓	✓	✓			
	PSO	^17^	✓	✓	✓			✓
	Simulation-based LOB (Learning/Forgetting + Crew Routing)	^44^	✓	✓	✓	✓		✓

Mathematical models—including linear, non-linear, constraint-based, dynamic, and fuzzy dynamic programming— provide explicit problem formulations with clearly defined objectives, decision variables, and constraints. While they yield exact solutions for small, well-structured cases, their scalability is limited, particularly when uncertainty, learning effects, interruptions, or multi-crew deployment are present ^15,21,29–37^.

Heuristic approaches rely on practitioner-defined sequencing rules, crew assignments, and activity start times. They are computationally efficient and simple to implement but often case-specific, producing near-optimal rather than truly optimal solutions ^38,39^. Well-known examples include LOB and resource-driven scheduling methods.

Metaheuristics—including Genetic Algorithms (GA), fuzzy GA, and Particle Swarm Optimization (PSO)—can efficiently search large solution spaces and model multi-crew deployment, learning effects, and soft logic ^17,20,40–43^. Despite their flexibility, they require careful tuning and may lack transparency for practitioners.

Recently, Hegazy et al. (2025) ^44^ introduced a simulation-based LOB approach in AnyLogic that integrates learning and forgetting effects into crew routing. Their model captures productivity losses due to reassignment, illustrating the trade-off between project duration and crew numbers and offering a scalable scheduling tool for large repetitive projects. However, while their ABM-based framework models routing and productivity evolution, it relies on heuristic rules and does not incorporate metaheuristic search, GIS-referenced spatial allocation, or stochastic replication. Their model also targets structured, linear repetitive settings, whereas SARPs involve non-sequential, geographically discontinuous units and shared contractor capacity across a distributed network.

The present research advances beyond Hegazy et al. (2025) by introducing several methodological enhancements. First, rather than relying on fixed production rates or pre-structured continuity rules, the proposed framework embeds decision-making within autonomous contractor and site agents operating in a GIS-linked environment, allowing routing decisions, idle gaps, and continuity patterns to emerge dynamically from micro-level interactions. Second, the framework adopts a spatially explicit nearest-feasible dispatching mechanism that captures travel-dependent behavior and represents geographic dispersion more directly than prior ABM formulations. Third, Monte Carlo replications quantify variability in makespan and in associated operational indicators (e.g., travel share and utilization), providing statistically robust performance evidence. Fourth, the framework is demonstrated on a mega-scale project, showcasing spatial heterogeneity and scale not reported in previous studies. Finally, the framework couples a GIS-enabled ABM with a GA-based optimization layer that enables exploration of improved contractor–site allocation and sequencing configurations through repeated simulation-based evaluation under stochastic conditions.

Recent work has also integrated GIS data into optimization frameworks to improve spatial realism. Gad et al. (2022) ^45^ developed a GIS-enabled SRP model that includes traffic patterns and site layouts, though it lacked shift-based planning and adaptive learning. Hegazy and Kamarah (2022) ^16^ introduced a GA-based desktop tool for distributed staff scheduling, but its scalability is limited by the absence of stochastic features such as uncertainty, learning curves, and real-world adaptability. Given the stochastic nature of SRPs, researchers have increasingly adopted Monte Carlo simulation and probabilistic buffers to model uncertainty ^46–49^. These methods enhance schedule resilience and improve mobile resource utilization. Other studies have incorporated buffer-time methods and learning effects to strengthen metaheuristic scheduling ^20,43^.

**Fig. 1:**
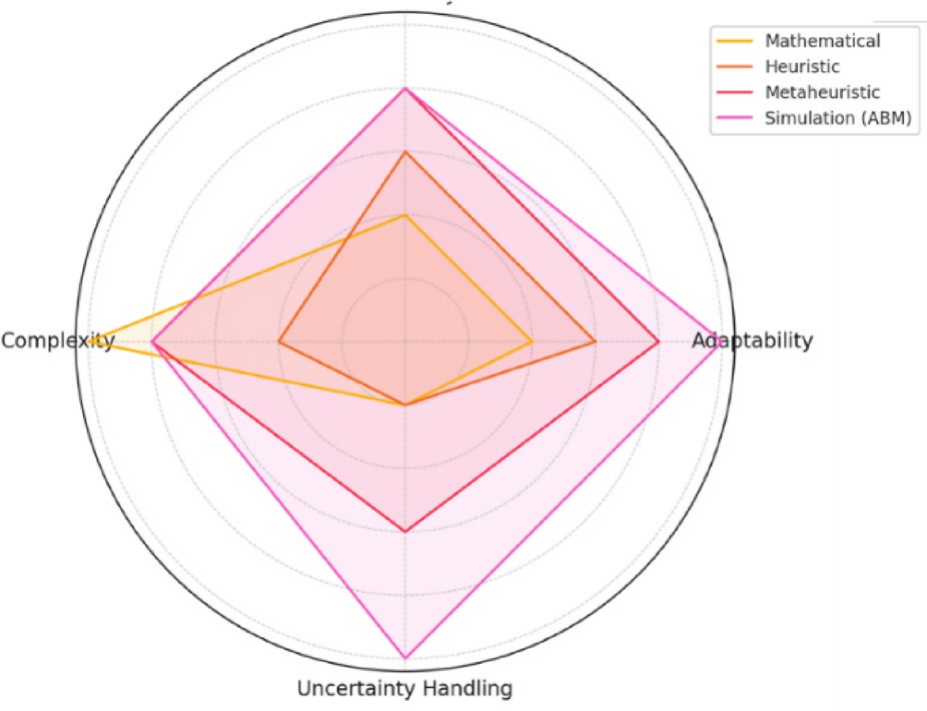
Qualitative trade-offs among mathematical, heuristic, metaheuristic, and simulation/ABM approaches across adaptability, uncertainty handling, and complexity.

A comparative overview of optimization streams—mathematical, heuristic, metaheuristic, and simulation-based—is shown in Fig. 1. The radar chart illustrates that simulation approaches, particularly ABM, offer superior scalability, adaptability, geographic flexibility, and uncertainty handling, whereas mathematical and heuristic models struggle in dynamic, large-scale environments. This reinforces the rationale for simulation-driven planning in SRPs.

**Fig. 2:**
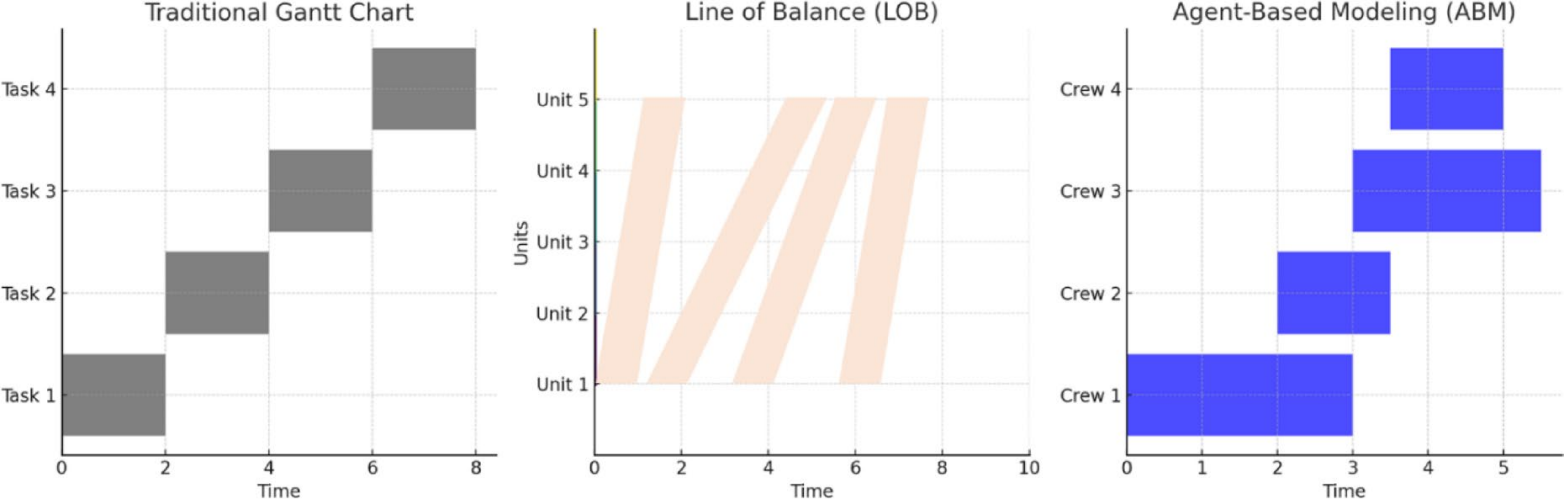
Comparison of Gantt, LOB, and ABM views of repetitive scheduling, emphasizing how each represents sequencing and crew continuity.

Figure 2 highlights conceptual distinctions between Gantt charts, LOB diagrams, and ABM for repetitive project scheduling. Whereas Gantt charts depict sequential durations and LOB charts illustrate workflow progression, ABM enables decentralized, real-time decision-making reflective of SARPs’ operational complexity. This flexibility allows ABM to better capture crew interactions, spatial routing, and dynamic resource allocation.

Commercial tools such as Primavera and MS Project have recently added repetitive scheduling templates, yet they do not natively support Agent-Based Simulation (ABS), stochastic modeling, or decentralized crew planning ^50–53^. Despite advancements in SRPs optimization, several gaps persist: (i) limited representation of SARPs in mainstream research, (ii) absence of organizational-level models for multi-crew deployment, (iii) underuse of ABM, (iv) weak treatment of uncertainty, and (v) lack of scalable tools for dynamic, scattered environments. Developing a unified, executable planning framework that jointly captures multi-crew deployment, spatial routing, and stochastic disruptions for SARPs remains limited in the literature.

Accordingly, this study contributes a planning approach that integrates GIS-enabled ABM with stochastic simulation for SARPs and couples the simulation with a GA-based optimization layer that enables exploration of makespan-oriented resource-allocation and sequencing configurations under multi-resource, sequencing/readiness, and spatial feasibility constraints. Validated using a real-world case study and multiple benchmark problems, the framework demonstrates how decentralized agent behavior, geographic routing, and uncertainty jointly shape schedule performance. In addition to makespan, operational indicators such as travel time/share, idle time, utilization, and continuity are reported as simulation-derived evaluation metrics to interpret deployment behavior and efficiency.

## Research methodology

This study adopts a structured four-phase methodology to develop, implement, and validate an ABS framework for multi-resource-constrained scheduling of SARPs. The process integrates spatial data, stochastic modeling, scenario testing, and statistical analysis to ensure methodological rigor and practical relevance.

**Phase I—problem identification and literature exploration**

This phase involved critically reviewing the state of knowledge in repetitive-project scheduling. Prior research on mathematical optimization, heuristic rules, and metaheuristics highlighted their limitations when applied to SARPs—projects characterized by dispersed locations, heterogeneous work scopes, and competing multi-crew demands. Industry consultations confirmed that traditional tools such as CPM, LOB, and commercial scheduling software perform poorly in environments requiring dynamic sequencing under spatial and uncertainty constraints.

**Phase II—research scope definition**

The scope was defined for modeling construction programs that involve multiple geographically dispersed sites—such as telecom towers, solar installations, housing clusters, and distributed utility networks. These projects require planners to manage: (i) crew continuity and limited contractor capacities, (ii) strict precedence relationships, (iii) travel-dependent routing decisions, and (iv) event-driven uncertainty. The study focuses on resource allocation and sequencing decisions to minimize total project duration (makespan) under feasibility constraints (capacity, precedence/readiness, and spatial dispersion). Travel time/share and crew idle time are recorded as emergent performance metrics produced by the simulation and used to evaluate operational efficiency and continuity behavior; they are not explicitly optimized. Explicit monetary costs are excluded and treated as future extensions.

**Phase III—model development**

This phase focused on developing a GIS-enabled ABS–optimization framework. Contractors and sites are modeled as autonomous agents operating in a spatial environment. Contractor agents make decentralized runtime decisions—such as when to request a new assignment and which feasible site to execute next—while site agents manage status transitions, precedence/readiness conditions, and disruption-induced delays. Runtime routing follows a nearest-feasible dispatching heuristic: after completing a site, each contractor selects the next unfinished, specialization-compatible site that minimizes travel distance among eligible alternatives. This policy is intended to promote continuity and limit avoidable travel, while overall performance is evaluated at the program level.

To search beyond a single heuristic run, the framework is coupled with a GA–based optimization layer that iteratively explores alternative contractor–site allocation and sequencing configurations by repeatedly executing the simulation and retaining improved solutions with respect to the makespan objective. Uncertainty is represented through probabilistic disruption events, sampled delay durations, and variable processing times enabling replication-based (Monte Carlo) performance assessments.

**Phase IV—model validation and scenario testing**

Model validation was conducted using a real-world telecom deployment program supplemented by four benchmark problems from literature, providing diverse testbeds in terms of spatial layouts and contractor capacities. Performance was assessed under both deterministic and stochastic conditions. Deterministic experiments established a baseline reflecting conventional practitioner heuristics, while the stochastic scenario—representing the primary experimental setting—incorporated risk events, processing-time variability, and routing behavior. For statistical rigor, random seeds were re-enabled in the simulation experiment to ensure non-repetitive stochastic behavior across runs. Monte Carlo replications were executed, each generating a full trace of makespan (project duration) and associated operational performance metrics (e.g., travel time/share, idle time, and construction time). For every run key performance indicator, mean values, standard deviations, and 95% confidence intervals (CI) were computed, producing narrow confidence bands that confirmed the stability and robustness of makespan reductions under probabilistic variability. Compared with the practitioner’s deterministic Excel plan (based on a basic CPM workflow), the proposed framework consistently achieved shorter makespans; idle time and travel share were reported as measured outcomes that also showed improved operational profiles under the proposed dispatching/assignment logic.

Replication-level results were summarized across Monte Carlo runs, with the full dataset provided in (Monte Carlo Supplementary Table S2). Sensitivity analyses were additionally performed by varying risk probability and contractor capacity to examine their influence on schedule performance and emergent system behavior.

## Problem statement and formulation

### Problem statement

SARPs involve multiple geographically dispersed sites, heterogeneous work scopes, and several mobile contractors (crews) with different specializations and capacities. The planning challenge is to determine: (i) which contractor executes which site, (ii) in what sequence, and (iii) at what start times, while accounting for spatial travel, contractor specialization, and stochastic delays.

## Research objective

The primary objective of this study is to address the SARPs resource allocation problem by minimizing the overall project duration (makespan) under crew specialization and availability constraints, together with sequencing/readiness and spatial feasibility considerations. Task continuity is promoted through the assignment/dispatching logic, and idle time, travel time/share, utilization, and continuity indicators are reported as emergent performance metrics produced by the simulation to evaluate operational efficiency and explain schedule behavior for decision support; they are not explicitly optimized in the objective function. The study therefore provides a practical, executable planning approach for dispersed, resource-constrained programs and evaluates its performance using replication-based evidence.

### Problem formulation

The scattered-site scheduling problem considered in this study consists of assigning a heterogeneous set of contractors $c\in C$ to a set of spatially distributed sites $j\in S$, with the objective of minimizing the overall makespan subject to feasibility constraints (assignment, qualification, capacity, and temporal feasibility).

#### Sets and indices

- $S=\{\mathrm{1,2},\dots ,n\}$: Set of construction sites
- $C=\{\mathrm{1,2},\dots ,m\}$: Set of contractors
- $$j\in S$$: Site index
- $$c\in C$$: Contractor index

#### Input parameters

- ${d}_{j}$*:* Base construction duration of site $j$*.*
- $lo{c}_{j}=({\lambda }_{j},{\phi }_{j})$: Coordinates of site $j$(latitude–longitude).
- $ca{p}_{c}$: Capacity of contractor $c$(maximum simultaneously active crews).
- $typ{e}_{j}$: Type of site $j$(e.g., Greenfield, Rooftop, Sharing).
- $specialization{s}_{c}$: Set of types of sites contractor $c$ is qualified to execute.
- $depo{t}_{c}$: Initial location of contractor $c$.
- ${D}_{a,b}$: Road-network travel distance between locations $a$ and $b$.
- $p$: probability that a disruption (risk event) occurs at a site (user-defined).
- $\Delta$: delay-impact model (user-defined), e.g., $Uniform\left[a,b\right]$ days.
- ${\delta }_{j}^{\left(r\right)}$: stochastic delay at site $j$ realized in replication $r$, generated by the risk model defined by $p$ and $\Delta$.

#### Decision variables

- ${x}_{c,j}\in \{\mathrm{0,1}\}$: equals 1 if contractor $c$ is assigned to site $j$, and 0 otherwise.
- $s{t}_{j}\ge 0$: start time of site $j$.

**Derived schedule quantity**

- $f{t}_{j}^{\left(r\right)}$: finish time of site $j$ in replication $r$, defined as:
  $$f{t}_{j}^{\left(r\right)}=s{t}_{j}+{d}_{j}+{\delta }_{j}^{\left(r\right)}$$

In deterministic settings, ${\delta }_{j}^{\left(r\right)}=0$ for all $j$. Under stochastic evaluation, ${\delta }_{j}^{\left(r\right)}$ is generated for each site $j$ within each replication $r$ according to the user-defined $p$ and $\Delta$.

#### Objective function

$$min {T}_{max}^{\left(r\right)}$$

subject to:

$${T}_{max}^{\left(r\right)}\ge f{t}_{j}^{\left(r\right)} \forall j\in S$$

The objective is to minimize the replication-level makespan ${T}_{max}^{\left(r\right)}$ , where $f{t}_{j}^{\left(r\right)}$ incorporates the deterministic duration ${d}_{j}$ and the replication-specific stochastic delay ${\delta }_{j}^{\left(r\right)}$. Other operational indicators (e.g., travel time/share, idle time, utilization, and continuity) are simulation-derived performance metrics used for evaluation and interpretation, but they are not explicitly included in the objective function.

#### Constraints

**Site assignment constraint**

Each site must be executed by exactly one contractor:

$$\sum_{c\in C}{x}_{c,j}=1 \forall j\in S$$

**Contractor specialization constraint**

A contractor may only execute sites that match its qualifications:

$${x}_{c,j}=0 if typ{e}_{j}\notin specialization{s}_{c} \forall c\in C,\forall j\in S$$

**Capacity constraint**

Contractor $c$ cannot exceed its simultaneous work capacity:

$\sum_{j\in S}active(c,j,t)\le {cap}_{c} \forall c\in C,\forall t$Here, $t$ denotes time in the scheduling horizon (continuous or discretized), and $active\left(c,j,t\right)$ is an indicator that equals 1 when contractor $c$ is executing site $j$ at time $t$, and 0 otherwise.

**Temporal feasibility**

Start times must be non-negative: $s{t}_{j}\ge 0, \forall \mathrm{j}\in \mathrm{S}$

#### Interpretation of the optimization problem

The SARP planning problem can be viewed as a spatially constrained, multi-crew, multi-resource-constrained project scheduling problem variant extended with: (i) geographic travel dependencies, (ii) contractor specialization requirements, (iii) heterogeneous site types, and (iv) stochastic execution times. The formulation above defines the objective (makespan minimization) and feasibility constraints (assignment, qualification, capacity, and temporal feasibility) in a method-agnostic manner.

At program scale, the system is dynamic and state-dependent: spatial routing, disruption realizations, resource competition, and decentralized decisions interact over time, making a single closed-form solution approach difficult to apply.

### Solution approach and simulation logic

This subsection describes the runtime decision logic used to operationalize the formulation within the GIS-enabled ABS environment. The elements below belong to the solution method (implementation/algorithmic logic) and are therefore separate from the method-agnostic problem formulation presented in Sect. "Problem Formulation".

#### Dispatching rule (nearest eligible site)

Upon completing its current site, contractor $c$ selects the next site based on a deterministic nearest-feasible heuristic: ${s}_{next}\left(c\right)=\mathrm{arg}\underset{j\in {S}_{c}^{ feasible}}{\text{ min}}{ D}_{(prev\left(c\right),\hspace{0.17em}j)}$

Where:

$${S}_{c}^{\mathrm{feasible}}=\{j\in S\mid eligible(c,j)=true{\hspace{0.25em}\hspace{0.05em}}\wedge {\hspace{0.25em}\hspace{0.05em}}unfinished(j)=true\}$$

**Symbol definitions**

- $prev(c)$: location of contractor $c$ after finishing its previous site.
- ${D}_{prev\left(c\right),j}$: travel distance from contractor $c$’s last location to candidate site $j$.
- $eligible(c,j)$: contractor $c$ is qualified to execute site $j$.
- $unfinished(j)$: site $j$ is not completed yet.

Here, $eligible\left(c,j\right)$ is determined by contractor specialization ($typ{e}_{j}\in specialization{s}_{c}$), and $unfinished\left(j\right)$ indicates sites not yet completed in the simulation state. If no feasible site exists, then $s_{{{\mathrm{next}}}} \left( c \right) = \emptyset$ and the contractor transitions to the *Idle* state. Accordingly, ${s}_{next}\left(c\right)$ is the output of the dispatching policy executed during the simulation.

#### Contractor continuity (field-realistic rule)

If a feasible site exists for contractor *c* (unfinished and eligible), the contractor should not remain idle:

$$\mathrm{If}: \exists j\in S : unfinished(j)=true{\hspace{0.25em}\hspace{0.05em}}\wedge {\hspace{0.25em}\hspace{0.05em}}eligible(c,j)=true$$

$$\mathrm{then}: contracto{r}_{state\left(c\right)}\ne idle$$

This rule reflects practical continuity expectations in mobile deployment operations and is implemented as part of the contractor agent’s runtime behavior.

#### Stochastic realism and monte carlo evaluation

Site durations incorporate random disruptions to embed uncertainty in processing time, enabling Monte Carlo analysis:

$${d}_{j}^{eff}={d}_{j}+{\delta }_{j}^{\left(r\right)} {\delta }_{j}^{\left(r\right)}\sim DiscreteRiskModel(p,\Delta )$$

where $p$ is the disruption probability and $\Delta$ defines the delay-impact distribution. ${\delta }_{j}^{\left(r\right)}$ is generated per site $j$ within each replication $r$. Performance is evaluated across independent Monte Carlo replications, reporting makespan and simulation-derived operational metrics.

## Proposed model framework

In this section, the conceptual foundations of the proposed framework are presented. The framework is built around ABM, where contractors and construction sites are represented as autonomous decision-making agents. Contractor agents govern assignment requests, mobility, and execution decisions, while site agents manage readiness, precedence conditions, and completion status under uncertainty. These interactions are encoded through state-based logic that captures the temporal, spatial, and stochastic characteristics of SARPs. Within a GIS-enabled spatial representation, schedules are generated by bottom-up operational rules. The framework targets makespan minimization as the primary optimization objective, while operational characteristics—such as travel time/share, idle time, utilization, and continuity patterns—are recorded as simulation-derived performance metrics for evaluating and interpreting solution behavior rather than being explicitly included in the objective function. Assignment and sequencing are operationalized through a transparent nearest-feasible dispatching heuristic, consistent with field practice, and an optimization layer is used to systematically explore alternative allocation/sequencing policies and identify those that yield improved makespan performance under the stated feasibility constraints.

### Agent-based modeling (ABM)

ABM is well suited to SARPs because it captures decentralized decision-making, rule-based interactions, and localized behaviors in settings with mobile resources, stochastic disturbances, and spatial variability. As shown by Bonabeau (2002) ^54^ and Macal & North (2010) ^55^, simple local rules can generate complex system-level patterns. Conventional CPM or system-dynamics formulations are either too rigid or too aggregated to reflect real-time site logic, uncertainty, and geographic dispersion across many units. By representing contractors and sites as autonomous agents, ABM enables local decisions (e.g., selecting the next feasible site or triggering crew deployment) to generate emergent system-level outcomes such as makespan, idle time, and travel load—insights that are difficult to obtain from traditional models.

In construction settings, ABM commonly faces calibration complexity because agent-level inputs (e.g., trade crew configurations, productivity distributions, learning/rework effects, shift patterns, and travel/mobilization characteristics) are rarely logged with sufficient granularity, and productivity is often non-stationary due to access constraints, sequencing, congestion, inspections/RFIs, and material availability—so calibration to historical durations may inadvertently fit hidden drivers and limit transferability.

ABM may also exhibit equifinality, where different dispatching or assignment rules yield similar aggregate outcomes, meaning that a good overall fit does not uniquely validate the underlying policy logic. Results can be sensitive to spatial and logistics assumptions (e.g., workface readiness, access routes, interference/congestion representation) and to boundary conditions such as approval cadence or mobilization constraints. In addition, ABM can pose transparency/interpretability challenges because outcomes emerge from interacting rules, and scalability may become a practical constraint when many agents, sites, and Monte Carlo replications are required. These considerations motivate transparent reporting of assumptions, careful verification/validation, and reporting performance across stochastic replications when applying ABM to dispersed, resource-constrained programs.

### Identifying key simulation parameters

Development of the model required structured datasets that capture the spatial, temporal, and stochastic characteristics of SARPs. Five main categories were used:

- **Site data:** Includes the number of geographically dispersed sites (e.g., *n* = 50), with unique identifiers and coordinates. Heterogeneity is reflected by documenting site types (e.g., *x* = 5) and their attributes. For each type, expected durations and defining properties are recorded—for example, type A requiring 40 days and type B 60 days—ensuring variability is accurately represented.
- **Contractor data:** Each contractor has a unique identifier and dispatch location coordinates. Crew capacities are specified per site type (e.g., two sites of type A, four of type B, none of type C). Crews remain dedicated to a single site until completion, mirroring real-world operational continuity.
- **Project calendar:** Captures temporal constraints such as national holidays, working days per week (e.g., five), and daily working hours (e.g., eight), ensuring schedules reflect realistic labor patterns and productivity limits.
- **Stochastic risk data:** Incorporates uncertainty through risk probabilities (e.g., *P* = 20%) and risk impact which is represented as normally distributed delays (e.g., $\updelta$= 1–10 days). This enables assessment of variability and the impact of disruptions such as weather, access, or permit delays. *(During model formulation, 20% was used illustratively to demonstrate the risk mechanism; all validation scenarios used a 15% risk probability).*
- **Other parameters:** A user-defined average truck speed is used to convert GIS-based distances into travel times. This parameter can be tuned to different contexts while leaving the underlying model structure unchanged.

Together, these datasets provide a comprehensive input base that reflects the operational realities of SARPs while supporting scenario analysis and sensitivity testing.

### Modeling environment

AnyLogic was selected for its hybrid simulation capabilities (ABM/Discrete-Event/System Dynamics), Java flexibility, and built-in GIS support, in addition to its embedded optimization functionality. AnyLogic’s Optimization Experiment supports two built-in search engines—OptQuest and a Genetic optimization engine—allowing model decisions and parameter combinations to be explored through repeated simulation-based evaluation. The Genetic engine is evolutionary (GA-based), operating on a population of candidate solutions and using selection and variation mechanisms to iteratively improve solutions while maintaining diversity, whereas OptQuest provides a general-purpose black-box optimization routine ^56^. In this study, the embedded optimization capability was used to tune contractor–site allocations (and the resulting sequencing/routing patterns) under the primary objective of makespan minimization; travel share and idle time were recorded as simulation-derived evaluation metrics.

Following Borshchev (2013) ^57^, combining ABM with real-world data and rule scripting enables realistic, adaptable modeling of large infrastructure systems. Geo-referenced GIS data further enhance the model by: (i) providing road-distance–based travel estimates converted to time using an average speed assumption (no real-time traffic modeling); (ii) supporting proximity-aware allocation behavior; and (iii) enabling spatial analysis of clusters, routing, and workload density. Compared with spreadsheet-based CPM planning, which typically ignore geographic variation, this GIS-enabled simulation–optimization setup supports program-scale exploration of allocation strategies under spatial and operational constraints.

### Model logic

**Fig. 3:**
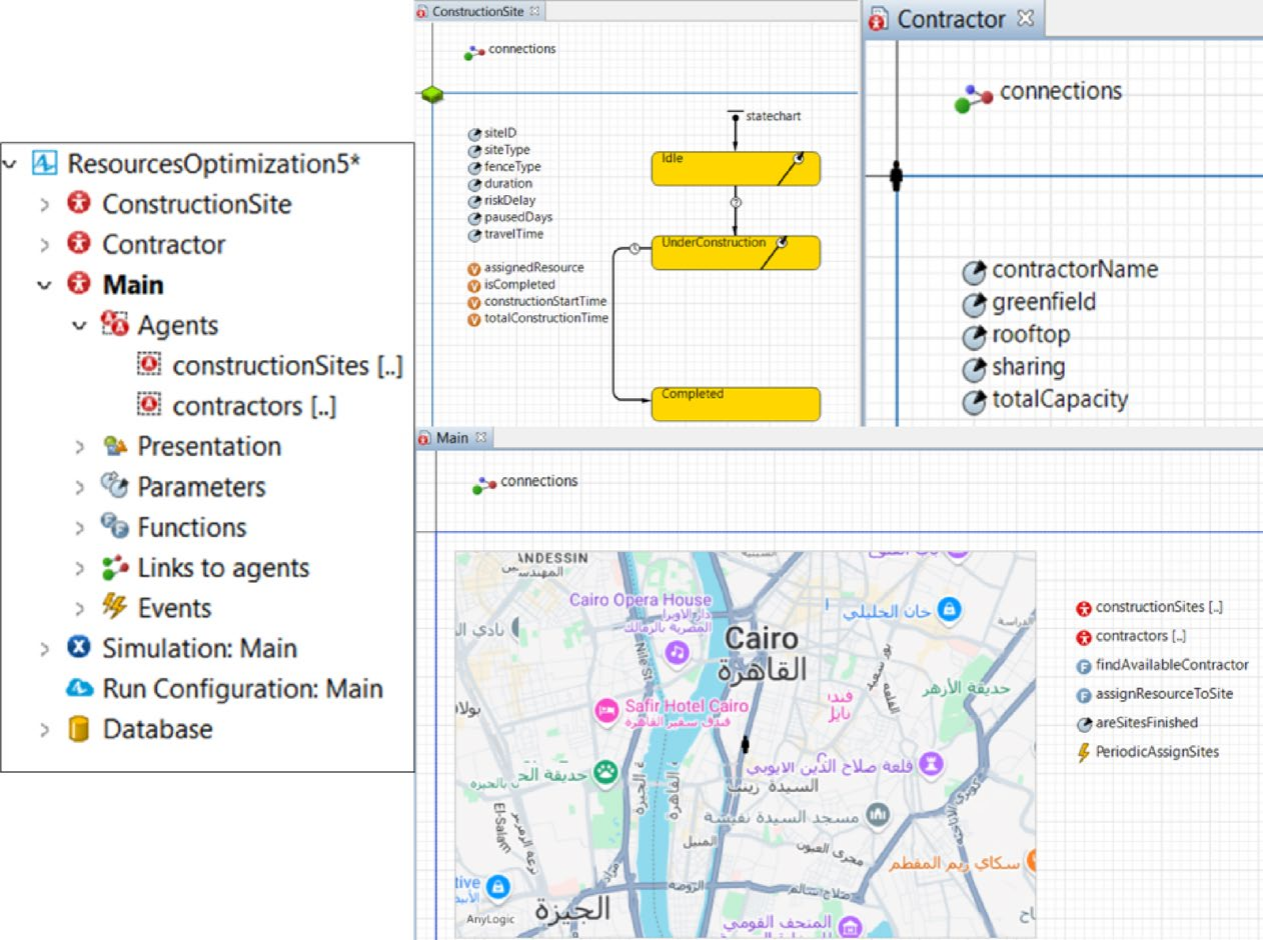
ABM implementation overview in AnyLogic (https://www.anylogic.com): agent classes (*Contractor*, *ConstructionSite*), state transitions, and GIS-based spatial representation used for routing and assignment (OpenStreetMap base layer: © OpenStreetMap contributors, ODbL 1.0; https://www.openstreetmap.org/copyright).

The overall model logic, implemented in AnyLogic, is summarized in Fig. 3. An OpenStreetMap base layer provides the geospatial framework for initializing construction sites and contractor depots.

The simulation framework is driven by three core components, each serving a distinct role: two agent types—*ConstructionSite* and *Contractor*—and a *Scheduler logic* unit:

- ***ConstructionSite*:** Models the spatial, structural, and temporal characteristics of each construction site.
- ***Contractor*:** Represents mobile construction crews responsible for executing tasks and moving between sites.
- ***Scheduler (logic unit)*:** Manages centralized task allocation, routing, and scheduling decisions across the agents.

In the simulation–optimization setup, the *Scheduler* is evaluated under alternative contractor–site allocation (and associated sequencing) configurations generated by the embedded optimization experiment. Each candidate configuration is assessed through repeated simulation runs, and the best-performing solution is selected with respect to the makespan objective, while operational metrics (e.g., travel share and idle time) are recorded for interpretation.

The primary assumption underlying the project schedule is that each site is assumed to be executed from start to finish by a single contractor without sharing crews, forming the basis for analyzing productivity, continuity, and crew efficiency across the program.

### Data integration initialization and key parameters

At runtime, the simulation imports two structured tables—*SiteData* and *ContractorsData*—which provide the attributes described in Sect. "Identifying Key Simulation Parameters". These files are attached to the model as database tables. Each record in *SiteData* is instantiated as a *ConstructionSite* agent, inheriting its type, duration, and coordinates. Likewise, each *ContractorsData* record becomes a *Contractor* agent with its specified depot and capacities. With two user actions—database attachment and agent population creation—the model is fully parameterized for any SARP, eliminating manual data entry and enabling rapid reconfiguration.

Sample input datasets are provided as Supplementary Materials to allow regeneration of the simulation environment, reproduce the reported experiments, and explore alternative scenarios.

#### Key simulation parameters

Key simulation parameters are then specified to capture core assumptions while allowing scenario tuning without altering the model structure:

- **Paused days**: Fridays were modeled as non-working days, reflecting a six-day labor calendar.
- **Travel speed**: A nominal average truck speed (e.g., 60 km/h) and an effective daily travel window (e.g., 8 h) are used to convert distances into travel days, making travel comparable with construction durations and delays.
- **Risk events**: Each site carried a probability of disruption (e.g., *P* = 20%), with impacts (δ) uniformly distributed between 1 and 10 days. These captured uncertainties such as weather, access or administrative interruptions.
- **Number of sites (*n*)**: User-defined *(e.g., 50 for illustrative experiments; 138 for the case study)*. The simulation incorporated 50 dispersed construction sites to reflect the repetitive, scattered nature of the project type under study.
- **Assignment logic**: Upon task completion, contractor routing follows a deterministic nearest-feasible heuristic: the contractor is dispatched to the closest eligible, unfinished site, based on GIS distance and specialization constraints.

This modular parameterization supports scalability, traceability, and robustness under varied logistical conditions.

### Agent interaction architecture

*Scheduler*, *Contractor*, and *ConstructionSite* agents communicate asynchronously through dynamic message exchange. While interactions are asynchronous, they remain coordinated: the *Scheduler* queries idle contractors and pending sites, matches them to the nearest feasible option, and updates their states. Separately, the GA-based optimization layer operates at the experiment level by varying candidate allocation/priority configurations across simulation runs and evaluating them against the makespan objective; it does not alter the agent messaging protocol described here. Once assigned, the *Scheduler* notifies the corresponding *ConstructionSite* agent, which then enters the *UnderConstruction* state. *Contractors* signal availability upon task completion, and sites signal readiness once prerequisites are met. This cycle continues until the global flag *areSitesFinished* evaluates true.

**Fig. 4:**
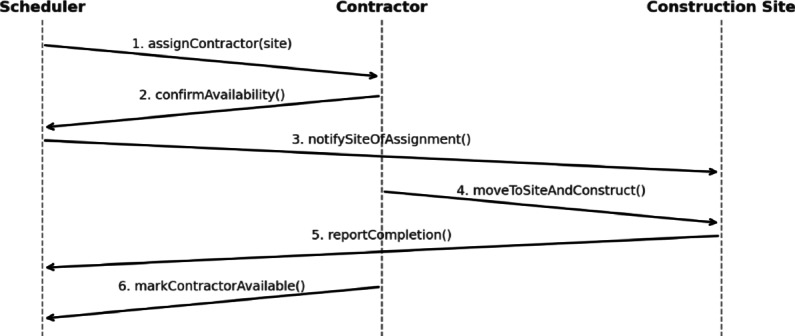
Agent interaction sequence implementing site assignment, availability checks, travel-to-site execution, and completion-driven redeployment.

Figure 4 depicts this interaction through a UML-style diagram. The architecture represents a centrally coordinated yet agent-driven framework: a single *Scheduler* governs task allocation, while local agents autonomously manage execution, routing, and state changes. This mirrors real-world practice in which centralized planning continuously adapts to site-specific constraints, specialized labor, and uncertain field conditions.

### *ConstructionSite* state-chart and scheduling algorithms

**Fig. 5:**
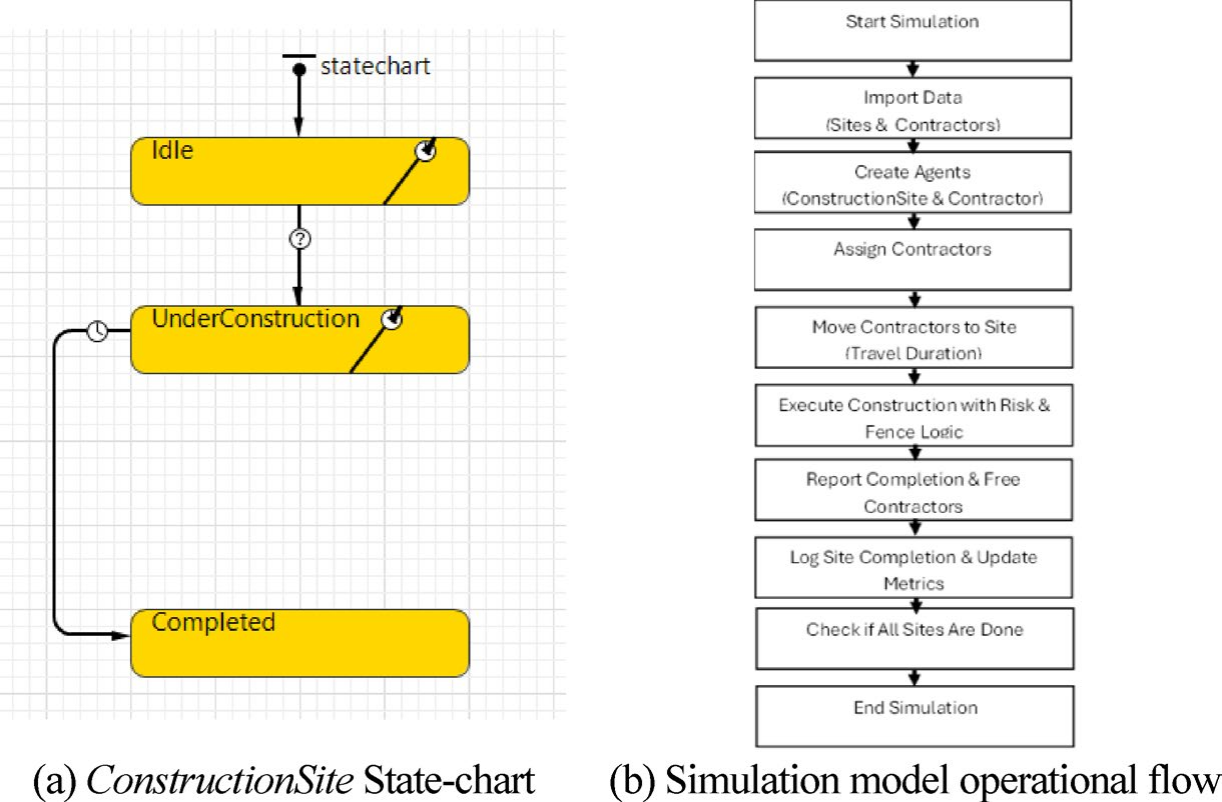
Unified state-chart and operational flow underpinning the ABM.

The lifecycle of each site is represented by a Finite State Machine (FSM) embedded in the *ConstructionSite* agent. As shown in Fig. 5a, the FSM contains three main states:

- ***Idle*:** The initial state where the site awaits contractor assignment.
- ***UnderConstruction*:** Active state; a contractor has been assigned, and construction is in progress. Site-type-dependent durations, stochastic risk delays, and non-working Fridays are all applied here.
- ***Completed*:** Terminal state; construction has finished, completion metrics are logged, and the contractor is released back into the available pool.

Figure 5b presents the overarching ABM workflow from data initialization to run termination, highlighting agent interactions, scheduling logic, and geospatial routing.

#### Transition triggers and java routines

Transitions between states are governed by timers and guard conditions that reflect real-world constraints, including travel, site variability, and probabilistic disruptions. The *Idle → UnderConstruction* transition is triggered when the *scheduler* identifies an eligible contractor based on specialization, proximity, and availability. This assignment-and-start routine is formalized in Supplementary Algorithm S1 (Supplementary Information file: *“Supplementary Algorithms S1”*): it (i) computes travel distance/time from the contractor’s current location using the selected distance method and working-time assumptions, (ii) applies stochastic disruption delays based on the specified risk parameters, and (iii) accounts for non-working days to convert effort into elapsed simulation time while logging key schedule events, ensuring that site start and execution timing reflect both spatial mobility and uncertainty in a parameter-controlled manner.

**Fig. 6:**
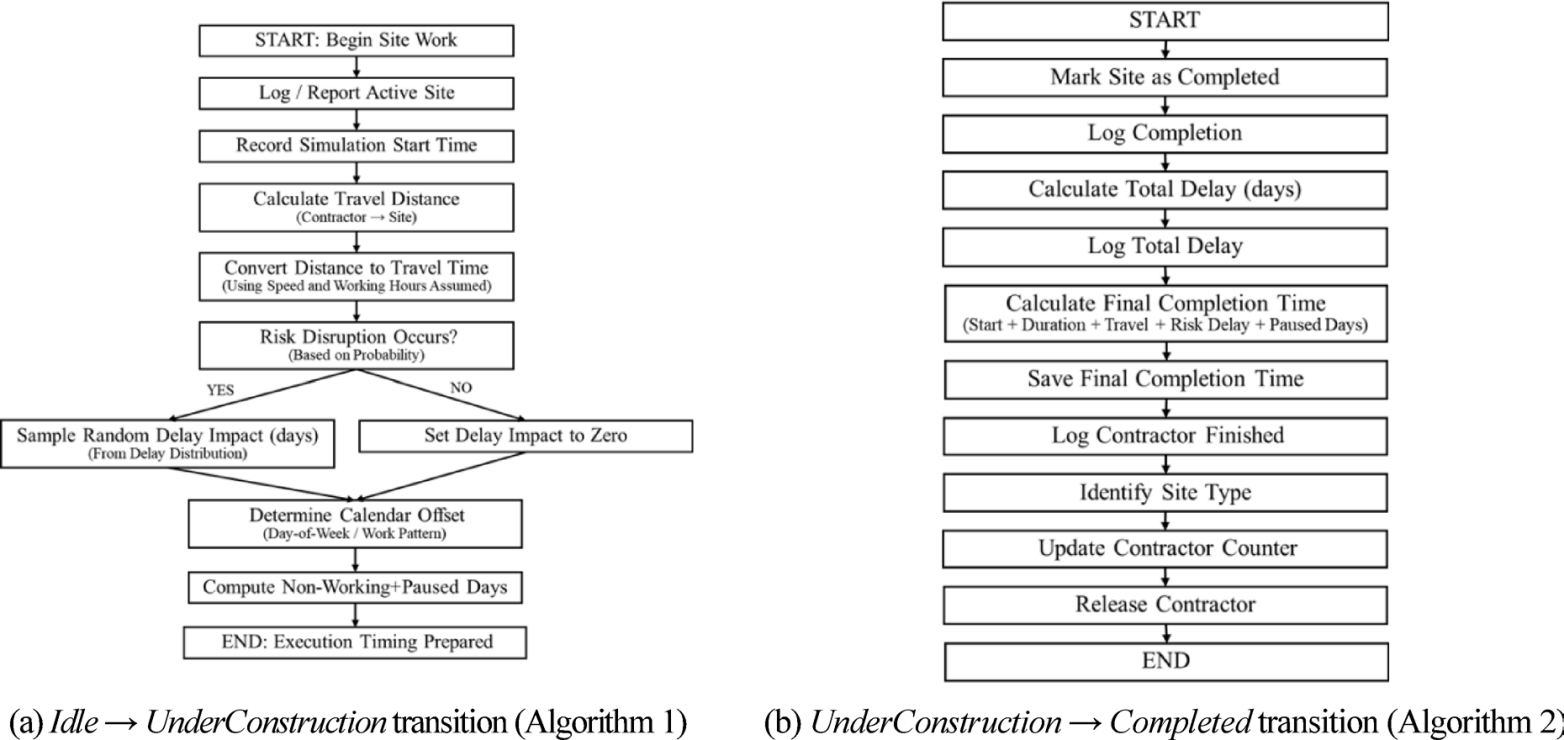
Simplified flowcharts of the two core state-transition routines used in the model.

The *UnderConstruction → Completed* transition is formalized in Supplementary Algorithm S2 (Supplementary Information file: *“Supplementary Algorithms S1”*): it (i) finalizes completion by marking the site as completed, (ii) computes and records the realized completion time by aggregating planned duration with travel time, stochastic disruption delays, and non-working-day pauses, and (iii) logs site-level delay components and updates contractor performance counters by site type before releasing the contractor back to the assignment pool. Figure 6a and b provide simplified visual flowcharts of the two routines.

#### Periodic scheduling routine

A recurring event, *PeriodicAssignSites*, serves as the operational scheduler, continuously scanning for idle contractors with the required specialization and assigning them to the nearest queued sites. The auxiliary functions *findAvailableContractor()* and *assignResourceToSite()* enforce specialization, capacity limits, and routing efficiency, while *areSitesFinished* controls replication termination. This modular, state-based logic provides transparent mapping between field operations and model behavior, supporting scenario testing and detailed evaluation of utilization, travel efficiency, and continuity under varying site mixes and risk settings.

### Key performance indicators (KPIs)

To evaluate system behavior and compare the proposed scheduling approach with baseline planning, a set of operational KPIs, summarized in Table 2, was computed directly from the simulation traces. These KPIs capture contractor-level, site-level, and program-level performance, including schedule efficiency, contractor utilization, routing effectiveness, and disruption impact.

**Table 2:** Key performance indicators (KPIs) used in the study.

KPI	Definition	Unit
Project duration	${T}_{max}^{\left(r\right)}=\underset{j\in S}{\mathrm{max}}f{t}_{j}^{\left(r\right)}$, where $f{t}_{j}^{\left(r\right)}=s{t}_{j}+{d}_{j}+{\delta }_{j}^{\left(r\right)}$	days
	Primary optimization objective; lower makespan indicates faster project delivery	
Contractor utilization (contractor $c$)	$\bigcup {til}_{C}=\frac{{BusyTime}_{C}}{{ActiveTime}_{C}}$	%
	Measures efficiency of contractor (*c*); higher utilization indicates tighter, more continuous workflows	
Idle time (contractor $c$)	${IdleTime}_{C}={\Sigma }_{t}\left(contractor\_state\left(c,t\right)=Idle\right)$	hours
	Time contractor (*c*) waits despite feasible assignments existing; indicates continuity gaps	
Construction time (contractor $c$)	${ConstTime}_{C}=\sum j:{x}_{c,j=1}{d}_{j}$	hours
	Time spent performing construction tasks (excluding travel and stochastic delay); measures productive effort	
Travel time (contractor $c$)	${TravelTime}_{C}= \sum \frac{{D}_{prev\left(c\right),j }}{{v}_{c}}$ (where ${v}_{c}$ = assumed travel speed)	hours
	Time spent moving between sites; lower values indicate tighter routing and less non-productive movement	
Travel distance (contractor $c$)	${TravelDist}_{C}= \sum {D}_{prev\left(c\right),j}$	Kilometers (km)
	Sum of GIS-measured distances travelled between consecutive site assignments; lower totals reflect more clustered routing	
Travel share (contractor $c$)	${TravelShare}_{C}= \frac{{TravelTime}_{C}}{{TravelTime}_{C}+ {ConstTime}_{C}}$	%
	Fraction of engaged time spent travelling rather than constructing; lower shares indicate higher productivity	
Active time (contractor $c$)	${ActiveTime}_{C }{=ConstTime}_{C}+ {TravelTime}_{C}$	hours
	Total engaged time (productive work plus travel) for contractor $c$	
Risk occurrence count	$RiskCount= {\sum }_{j\epsilon s}1 ({\delta }_{j}>0)$	count
	Number of sites where stochastic delays were triggered reflects disruption frequency	
Risk-induced delay	$RiskDelay= {\sum }_{j\epsilon s}{\delta }_{j}$	hours (or days)
	Cumulative additional time added to base durations due to risk events; measures schedule impact of uncertainty	
Sites-per-day throughput	$Sites per day= \frac{\left|S\right|}{{T}_{max}}$ where $\left|S\right|$ is the total number of sites	sites/day
	Emergent productivity rate of the overall system	
Average assignment distance (contractor $c$)	${AvgDist}_{C}= \frac{1}{{N}_{C}-1}\sum {D}_{prev\left(c\right),j}$ ${N}_{C}$ is the number of sites assigned to contractor *C*	kilometers (km)
	Mean GIS distance between consecutive tasks for contractor $c$; direct measure of routing efficiency	

### Model output: automated streams and user derived metrics

The agent-based model produces a comprehensive decision-support dataset through two complementary output streams:

Automated outputs (generated dynamically within AnyLogic):

- ***Console feed:*** Provides a detailed execution trace including contractor-site assignments, computed travel time and distance, construction start and finish events, risk triggers and sampled delays, total site-construction durations, and the final project makespan. Additional variables (e.g., queue lengths, state transitions) can be logged as required.
- ***Database logs:**** Agent Movement* (crew relocations with timestamps and kilometers), *Agent Movement Stats* (per contractor aggregates), *State-chart Stats* (entry/exit dates for *Idle*/*UnderConstruction*/*Completed*), *State-chart Time* (mean/minimum/maximum dwell times), and *State-chart Transitions* (timestamped triggers). The full AnyLogic schema is richer; only key tables are reported here. These logs enable granular, post-run diagnostics, providing day-by-day (or finer) progress.

(b) User-derived outputs (calculated from automated data):

- Analysts can compute the KPIs listed in Table 2 directly from these logs, including total active time, travel time, idle time, utilization, productivity (sites/day), per-contractor site counts, and identification of top/least performers. Spreadsheet or script-based processing can be used, leveraging the high-resolution traces for flexible post-hoc analysis.

Together, these outputs transform raw simulation runs into an actionable analytics package, equipping project managers with evidence to benchmark deployment policies, diagnose inefficiencies, and support iterative refinement of deployment policies and scheduling logic in subsequent cycles.

#### Stochastic replication protocol

To ensure statistical robustness, the agent-based model was executed using independent Monte Carlo replications, each with a different random seed. Processing times, risk delays, and travel variations were resampled in each run. For each KPI—project makespan and the associated operational indicators (idle time, travel time, active time, and travel share)—we computed the sample mean, standard deviation, and 95% confidence intervals (CI) using:

$$CI = \overline{x} \pm 1.96 \cdot \frac{s}{\sqrt n }$$

where $\overline{x}$ is the sample mean, $s$ is the sample standard deviation, and $n$ is the number of replications. This statistical treatment quantifies uncertainty in schedule outcomes and aligns with best practices in stochastic construction simulation. The narrow CI observed across key indicators confirm that the schedule compression results are consistent and robust under probabilistic variation.

## Model validation and verification

This section is organized into two key subsections: model validation and model verification. The model validation subsection will focus on testing the model against real-world data, assessing how well the model’s results align with actual outcomes to ensure its reliability and accuracy in practical applications. The model verification subsection, several iterations will be performed to ensure that the model functions as intended, confirming its accuracy and preventing any unexpected or anomalous outcomes.

### Illustrative case-study description

Mobile network towers host antennas, transmitters, and related equipment to maintain signal coverage and enable connectivity. Rollouts typically involve three tower types: Greenfield (GF), free-standing structures on land parcels; Rooftop (RT), lighter masts anchored to building roofs; and Sharing sites, where new equipment is co-located on existing GF or RT towers. While radio hardware is similar, construction sequences differ. GF towers require full civil works—from land handover and excavation through raft foundations, concrete casting, tower erection, and fencing. RT towers follow a similar process but omit fencing and substitute roof anchoring for excavation. Sharing sites bypass most civil works, limited to mounting brackets, antennas, and cabinets, making them fastest to deliver. Typical durations are 2–3 days for Sharing sites, 14 days for RT towers, 30 days for GF with steel fencing, and up to 40 days with brick fencing. The dispersed locations, varied time frames, and site-by-site crew allocation make mobile network tower programs archetypal SARPs, and thus an ideal test case for the proposed simulation framework.

#### Illustrative case‑study dataset and data collection

**Fig. 7:**
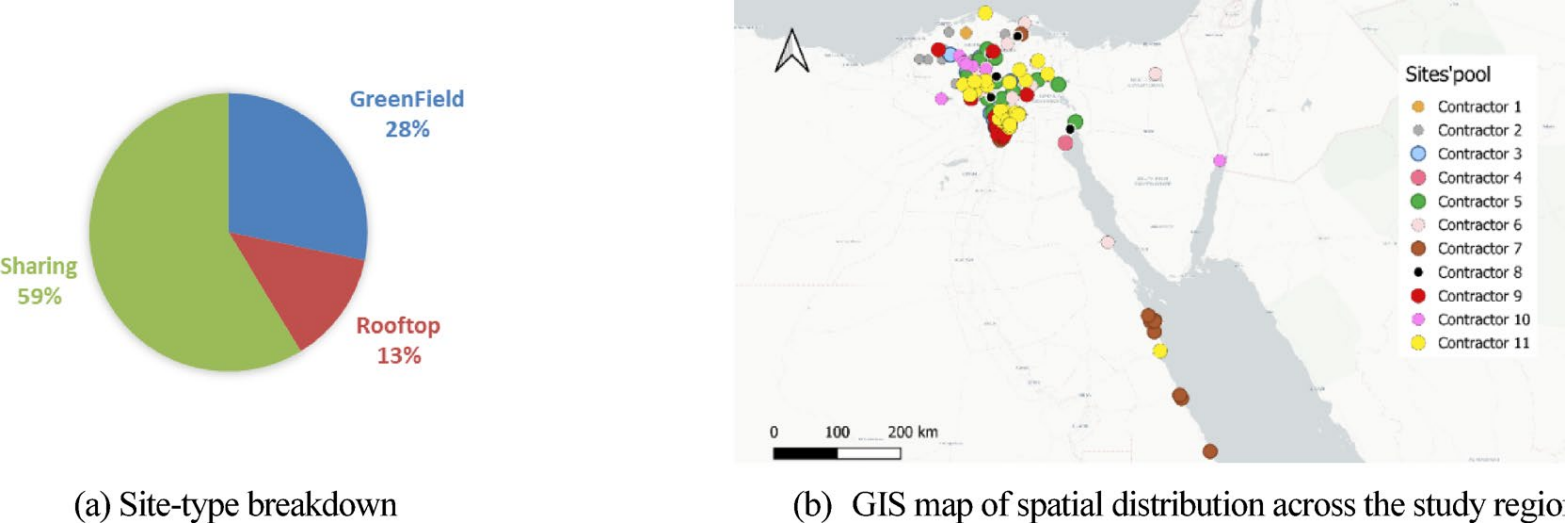
Input site pool for the case study: (**a**) site-type composition; (**b**) spatial distribution of the 138 sites. Map in (b) rendered in QGIS 3.36 (LTR) using OpenStreetMap tiles ( © OpenStreetMap contributors, ODbL 1.0; https://www.openstreetmap.org/copyright).

The case-study dataset originates from a real-world mega infrastructure project of mobile-network deployment program, comprising 138 tower sites distributed across Cairo, Sinai, and the Delta regions of Egypt. Raw records from the project’s vendor were cross-checked against as-built logs and cleaned to remove missing coordinates or duplicate identifiers. The final dataset comprises four structured components:

*a. Available sites pool* – Each site is assigned a unique Site ID, precise latitude–longitude coordinates, and site type. For all GF sites, fence type—brick wall or steel—was specified. The pool consists of three dominant site types: 59% Sharing, 28% GF, 13% RT (Fig. 7a). Typical base durations are ≈ 3 days for Sharing sites, 14 days for RT towers, and 30–40 days for GF sites depending on fence type. A GIS layer was created from the site coordinates and visualized in QGIS 3.36 (LTR) using OpenStreetMap tiles ©, which illustrates both the categorical mix and the broad geographic dispersion (Fig. 7b), supplying spatial inputs for the ABM routing engine.

**Fig. 8:**
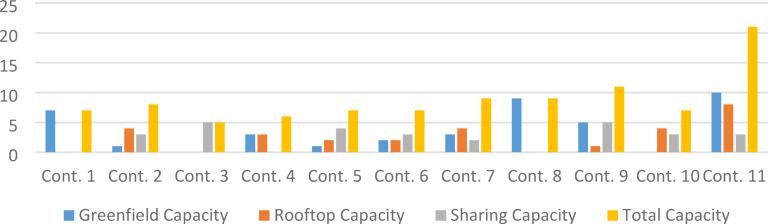
Heterogeneous contractor capacity across site types, used to represent multi-resource availability constraints in the case study.

*b.Contractors and crew capacities –* The contractor dataset lists all participating contractors, their home-base coordinates, and their type-specific crew capacities. Each contractor deploys autonomous crews that execute entire sites from mobilization to completion without substitution or handoff. Capacity is site-type dependent; for example, Contractor 10 can execute four RT and three Sharing sites concurrently but has no GF capability due to civil-works specialization. Table 3 summarizes the available capacities across GF, RT, and Sharing sites, and Fig. 8 visualizes these allocations, supplying both capacity and spatial constraints for the simulation.

**Table 3:** Available contractors’ capacities for base case study.

Contractor	Greenfield Capacity	Rooftop Capacity	Sharing Capacity	Total Capacity
Cont. 1	7	0	0	7
Cont. 2	1	4	3	8
Cont. 3	0	0	5	5
Cont. 4	3	3	0	6
Cont. 5	1	2	4	7
Cont. 6	2	2	3	7
Cont. 7	3	4	2	9
Cont. 8	9	0	0	9
Cont. 9	5	1	5	11
Cont. 10	0	4	3	7
Cont. 11	10	8	3	21

*c.* *Benchmark schedule –* The owner’s practitioner-generated Excel CPM-based schedule, documenting actual start and finish dates and observed crew movements, is used as the baseline heuristic plan for comparative evaluation of the simulation outputs*.*

*d.Dynamic parameters –* Travel times were computed using numerical outputs from the Google Maps Distance Matrix API. Only distance and duration values were used (no imagery), and no real-time traffic effects were incorporated. Reported durations were interpreted under an average-speed assumption consistent with the model’s travel-time formulation.

Together, these cleaned, multi-layer datasets supply the geographic, temporal, and resource capacity foundations for the agent-based, GA-driven optimization framework described in Sect. "Proposed Model Framework".

### Model validation

After importing the two primary datasets—*siteData* and *contractorsData*—into AnyLogic as database tables, the project’s spatial layout is immediately visualized on the GIS-enabled canvas. Each site is displayed as a geo-referenced marker, providing a clear overview of the network’s geographic dispersion across urban, peri-urban, and remote regions. This spatial distribution reflects the heterogeneity typical of SARPs and highlights the routing, accessibility, and capacity-allocation challenges that motivate an agent-based approach.

Following dataset import and visualization, the simulation environment was configured using parameters calibrated directly from the real deployment program. The project calendar reflects contractual conditions—a six-day workweek, Fridays as non-working days, and an eight-hour daily shift. Stochastic risk settings were likewise grounded in empirical data: during model development, a nominal 20% probability was used to illustrate delay propagation, while the validated scenarios adopted a calibrated 15% probability consistent with the frequency of delay-causing incidents recorded in historical rollout logs. Delay magnitudes were sampled from a uniform 1–7-day range, matching observed access, permitting, and material-delivery disruptions in the field. Travel durations were estimated using numerical outputs from the Google Maps Distance Matrix API for representative origin–destination pairs, yielding an average effective travel speed of approximately 60 km/h, which was used as the routing parameter. These calibrated inputs ensure that the simulation reproduces the operational and uncertainty conditions of the real-world case as closely as possible.

**Fig. 9:**
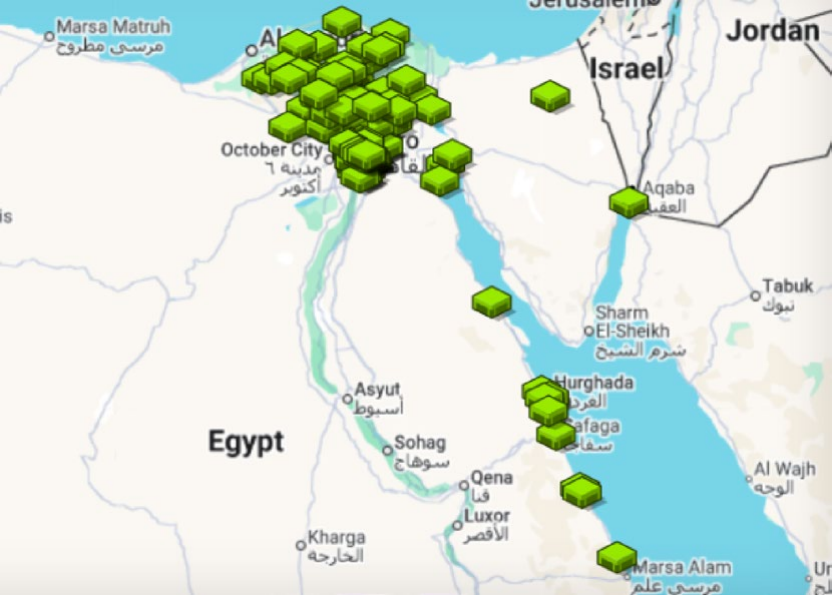
GIS-based spatial context of the case-study sites, enabling explicit modeling of dispersion and travel between sites. Map rendered in AnyLogic 8.8.x (https://www.anylogic.com) using OpenStreetMap tiles ( © OpenStreetMap contributors, ODbL 1.0; https://www.openstreetmap.org/copyright).

Figure 9 displays the spatial distribution of all active sites as rendered within the AnyLogic GIS environment (based on OpenStreetMap tiles©), illustrating the broad dispersion and geographic complexity incorporated into the model. To support reproducibility, sample of the input datasets used in this validation—*siteData* and *contractorsData*—are provided in the supplementary package.

#### Results analysis

**A. Comparison against field baseline**

The simulation output was benchmarked against the practitioner’s heuristic Excel CPM-based schedule, which estimated 100 days for program completion. Under identical crews and spatial constraints, the agent-based model consistently produced substantially shorter makespans.

Across 20 independent Monte Carlo replications—each with a unique random seed to vary travel times, risk events, and processing-time deviations—the project duration converged to a narrow range of 49–56 days, representing an average improvement of ≈46.25% over the field baseline.

These gains primarily result from the distance-aware assignment logic, which:

- Allocates idle contractors directly to the nearest feasible site,
- Minimizes non-productive travel, and
- Reduces idle-time clustering.

**B. Statistical robustness across replications**

Table 4 summarizes replication-level statistics. Key observations include:

- Small standard deviations across all KPIs.
- Tight 95% confidence intervals, indicating stable behavior.
- A mean percentage reduction of 46.25%.
- A 95% confidence interval of 45.44%–47.07%.

**Table 4:** Statistical Summary Across 20 Replications.

KPI	Mean ($\overline{x}$)	Std. Dev. (*s*)	95% CI
Duration (days)	53.75	1.86	52.93–54.56
Idle Time (hrs.)	14,800.8	298.45	14,669–14,931
Travel Time (hrs.)	74.77	9.14	70.77–78.78
Construction Time (hrs.)	54,547	444	54,352–54,742
Travel Share (%)	0.137	0.017	0.130–0.144

These patterns confirm that performance gains are systematic, not driven by outlier runs.

Monte Carlo Supplementary Table S2 provides complete replication-level datasets for reproducibility.

**C. Internal model validation**

Internal diagnostic checks confirmed that model behavior aligned with field operational expectations. Across all 20 replications:

- All sites received an initial contractor assignment by Day 17, even in the longest 55–56-day runs. This validates the early-allocation and continuity-preservation logic of the dispatching mechanism.
- Under a calibrated 15% risk-event probability, each replication exhibited:
-  ~ 21 sites affected by delays (on average),
- Correct propagation of delays into site-level durations,
- No violations of specialization or sequencing constraints.

**D. Representative contractor-level analytics**

**Fig. 10:**
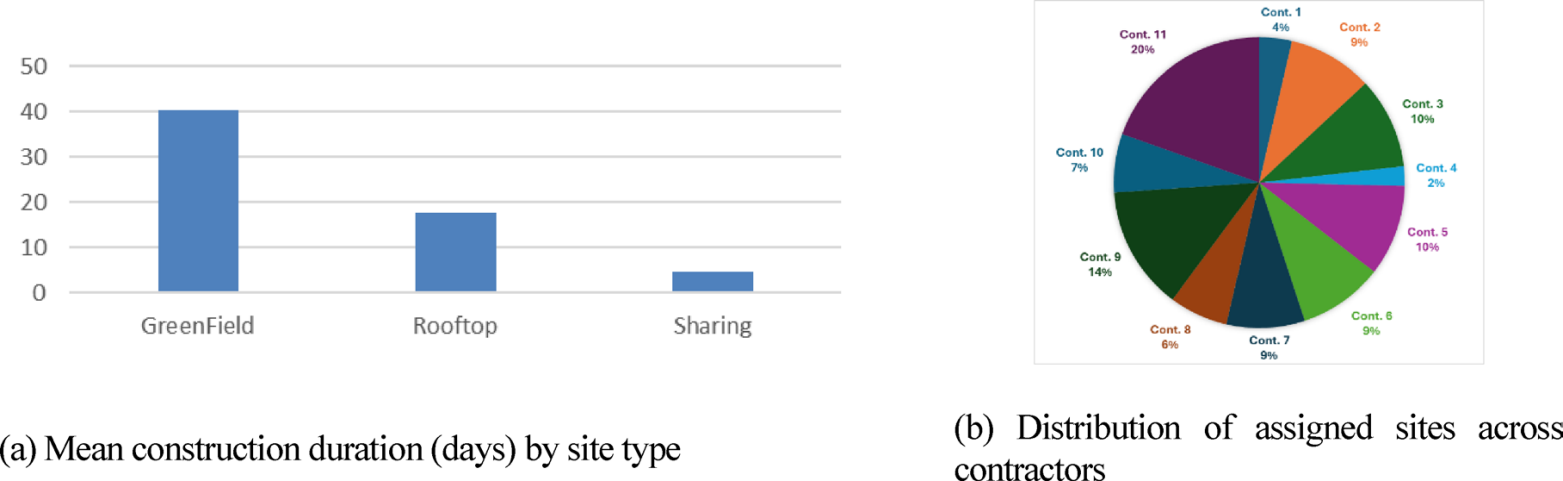
Site-type workload and allocation outcomes.

**Fig. 11:**
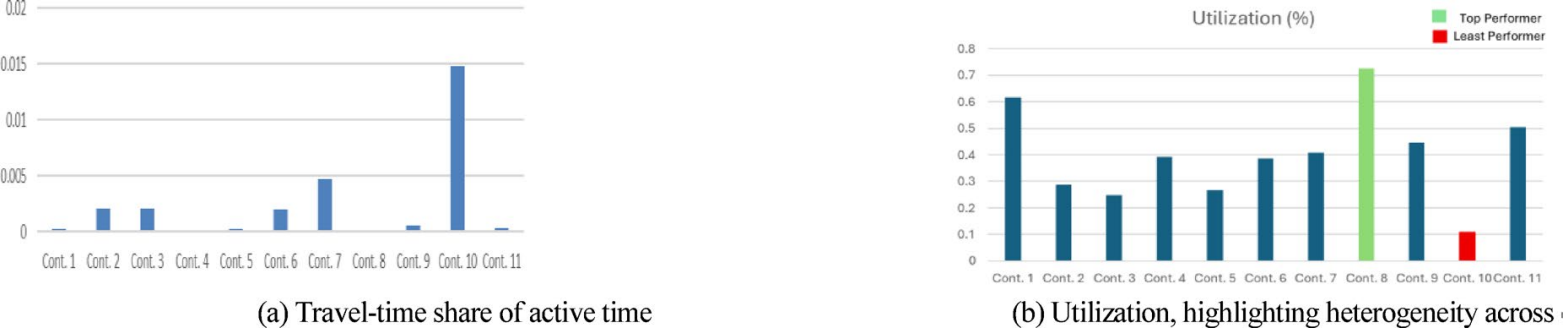
Contractor-level performance from a representative replication.

**Fig. 12:**
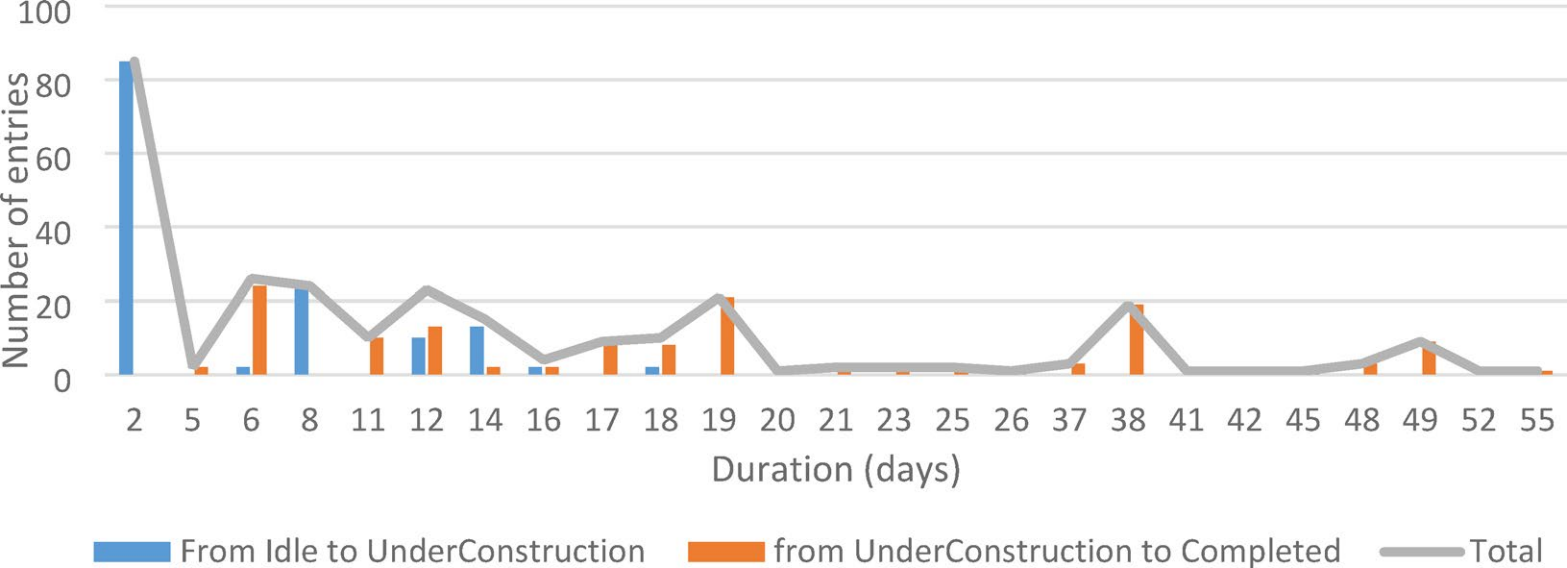
Event dynamics of the ABM schedule: daily transitions into *UnderConstruction* and into *Completed*, indicating program ramp-up and completion patterns.

Contractor-level analytics shown in Figs. 10–12 correspond to one representative replication that produced a 55-day makespan, selected because it fell near the sample mean and exhibited the typical stochastic structure observed across runs. These visuals therefore illustrate a representative behavioral profile rather than a single-run anomaly. Across the 20 Monte Carlo replications, contractor-level distributional patterns—such as utilization ranking, travel-share ordering, and the relative distribution of assignment loads—remained qualitatively stable. This stability allows a single run to serve as a clear, interpretable illustration without affecting the integrity of the aggregated numerical findings.

Figure 10 presents (a) mean durations per site type—capturing base duration, travel, risk delays, and Friday pauses—and (b) each contractor’s proportional share of total site assignments. Figures 11a–b highlight travel time as a share of active time and contractor utilization percentages, exposing underperforming or travel-burdened teams that would be difficult to detect under heuristic field planning. Figure 12 visualizes day-by-day contractor state *transitions (Idle → UnderConstruction → Completed)*, revealing continuity patterns, avoidance of prolonged idle clustering, and realistic workload distribution. Productivity rates in the representative run ranged from 0.09 to 0.49 sites/day, with a system-level emergent average of 0.228 sites/day.

Collectively, these diagnostics show that the ABM framework produces interpretable, monotonic responses to input variation, respects all routing and specialization constraints, and surfaces inefficiencies that cannot be captured using traditional heuristic scheduling. Computational performance remained practical: complete run times ranged from *63.98* to *155.26* s (base case: 89.1 s), enabling rapid scenario exploration and “what-if” testing during planning meetings.

#### Visual validation

**Fig. 13:**
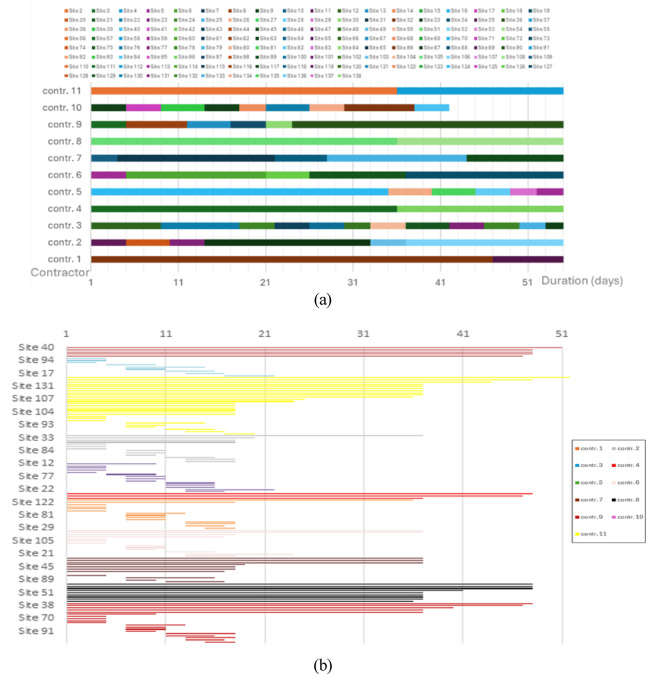
Continuity and program-level scheduling behavior from a representative stochastic replication (55 days). **(a)** Contractor activity timelines showing illustrating redeployment across sites with minimal idle gaps (visual evidence of continuity under the nearest-feasible assignment logic). **(b)** Project-level Gantt chart by site illustrating parallel execution across the program and resulting schedule flow.

Visual validation was used to confirm that the behavioral patterns implied by the KPIs are also evident in the temporal evolution of the schedule. Figures 13a–b presents two complementary views from a representative stochastic replication (55-day run). Although the manuscript reports all numerical results based on 20 independent Monte Carlo replications, contractor-level patterns (e.g., utilization ranking, travel-share ordering, continuity behavior) were qualitatively stable across all runs. Therefore, a single run is sufficient to visually demonstrate emergent behavior without affecting the statistical validity of the reported results.

Figure 13a (contractor activity timelines across the 55-day program) visualizes continuity and redeployment dynamics: each row corresponds to one contractor, and the near-uninterrupted sequences of colored segments indicate that contractors transition quickly from one feasible site to the next with minimal idle gaps. Short transitions between segments represent redeployments driven by the nearest-feasible assignment logic, while the limited fragmentation across each row provides visual evidence that the continuity rule avoids frequent stop–start cycles.

Figure 13b (project-level Gantt chart by site) summarizes the resulting scheduling behavior at the program level: parallel progress across many sites is evident from the dense horizontal activity early in the horizon, all 138 sites received an initial crew assignment by day 17, and the schedule tail is governed by the long-duration GF tasks. Importantly, the absence of gaps, or stranded and incomplete site segments, and the consistent progression of activity blocks support correct state transitions (*Idle → UnderConstruction → Completed*) and constraint enforcement (e.g., readiness and capacity), consistent with the observed reduction relative to the practitioner’s Excel CPM-based baseline.

These visual diagnostics complement the statistical analysis by showing how local contractor decisions (continuity-preserving redeployment) translate into coherent program-level flow and stable scheduling patterns under stochastic replications. This further demonstrates that the model behaves coherently at the operational level and that the emergent scheduling patterns align with field expectations.

#### Spatial assignment analysis

A spatial analysis was conducted to evaluate how closely the nearest-feasible assignment rule mirrored geographical proximity. The analysis was performed on the illustrative 55-day run used for visual reporting; however, the qualitative patterns (clustering strength, far-assignment frequency, contractor travel ordering) were consistent across the 20 Monte Carlo replications. To quantify spatial dispersion, a virtual centroid was computed for each contractor (mean latitude/longitude of all sites assigned to that contractor). The great-circle distance between each assigned site and its contractor’s centroid was then calculated, allowing the extraction of summary statistics—mean distance (μ), standard deviation (*s*), and maximum hop distance. Any site with a centroid distance greater than 200 km was flagged as a far assignment. Only four sites out of 138 (≈3%) exceeded this threshold (Table 5), confirming that the distance-minimizing heuristic generally maintained strong geographic clustering.

**Table 5:** Summary of centroid-based travel distance analysis.

Contractor	Sites count	Mean travel distance μ (km)	*s* (km)	Max distance (km)	Farthest site
Contractor 1	5	54.7	30.1	108.2	Site 35
Contractor 2	13	39.7	20.1	78.5	Site 11
Contractor 3	14	37.7	24	97.0	Site 43
Contractor 4	3	43.1	19.2	64.7	Site 57
Contractor 5	14	50.2	28.1	119	Site 81
Contractor 6	13	87.9	70.4	264.2	Site 62
Contractor 7	12	206.8	108.8	404.6	Site 10
Contractor 8	9	56.4	28.5	104.5	Site 106
Contractor 9	19	36.6	30.5	131.2	Site 67
Contractor 10	9	96.2	99.5	356.6	Site 39
Contractor 11	27	58.3	84.5	450.6	Site 92

**Fig. 14:**
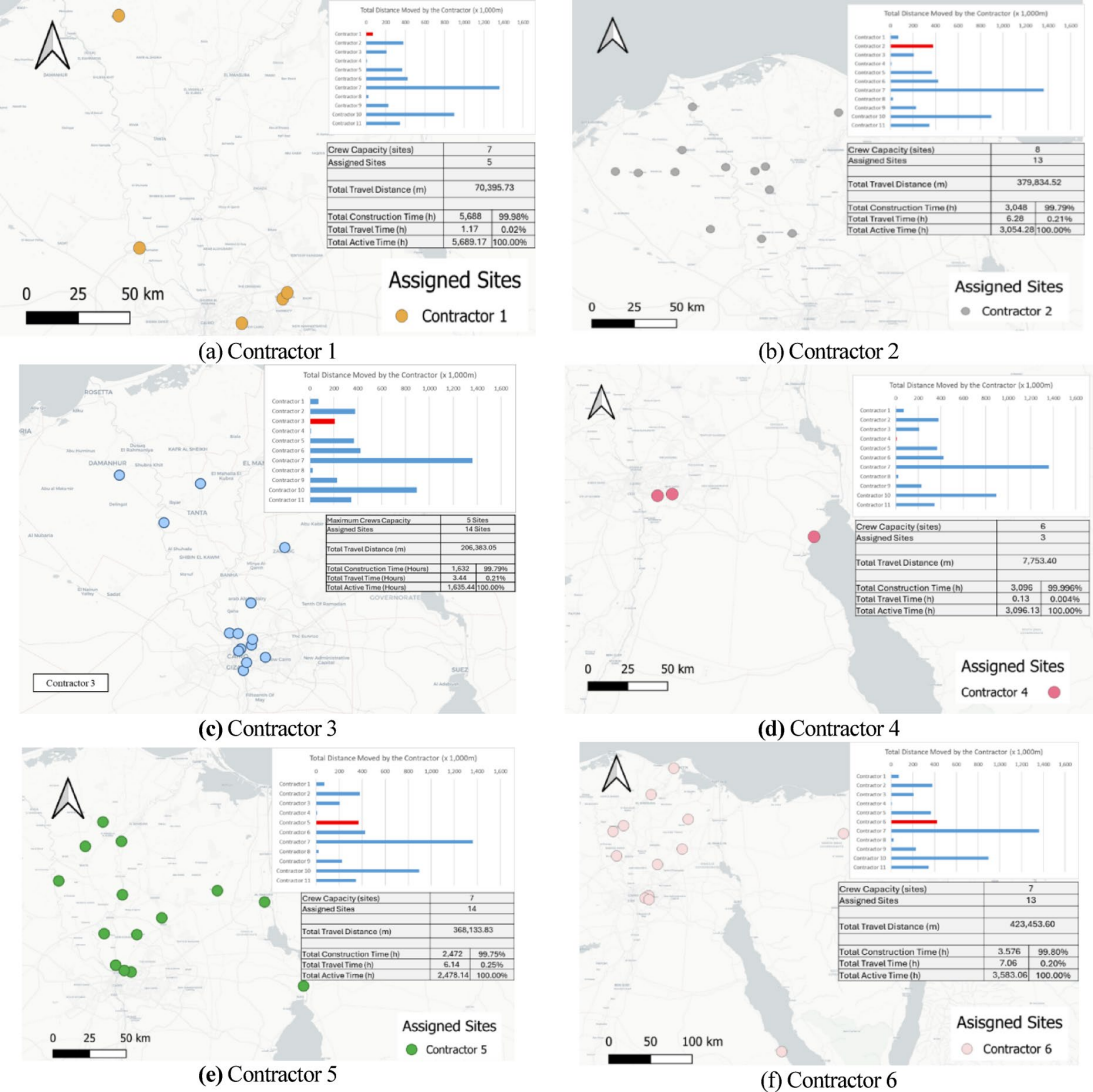

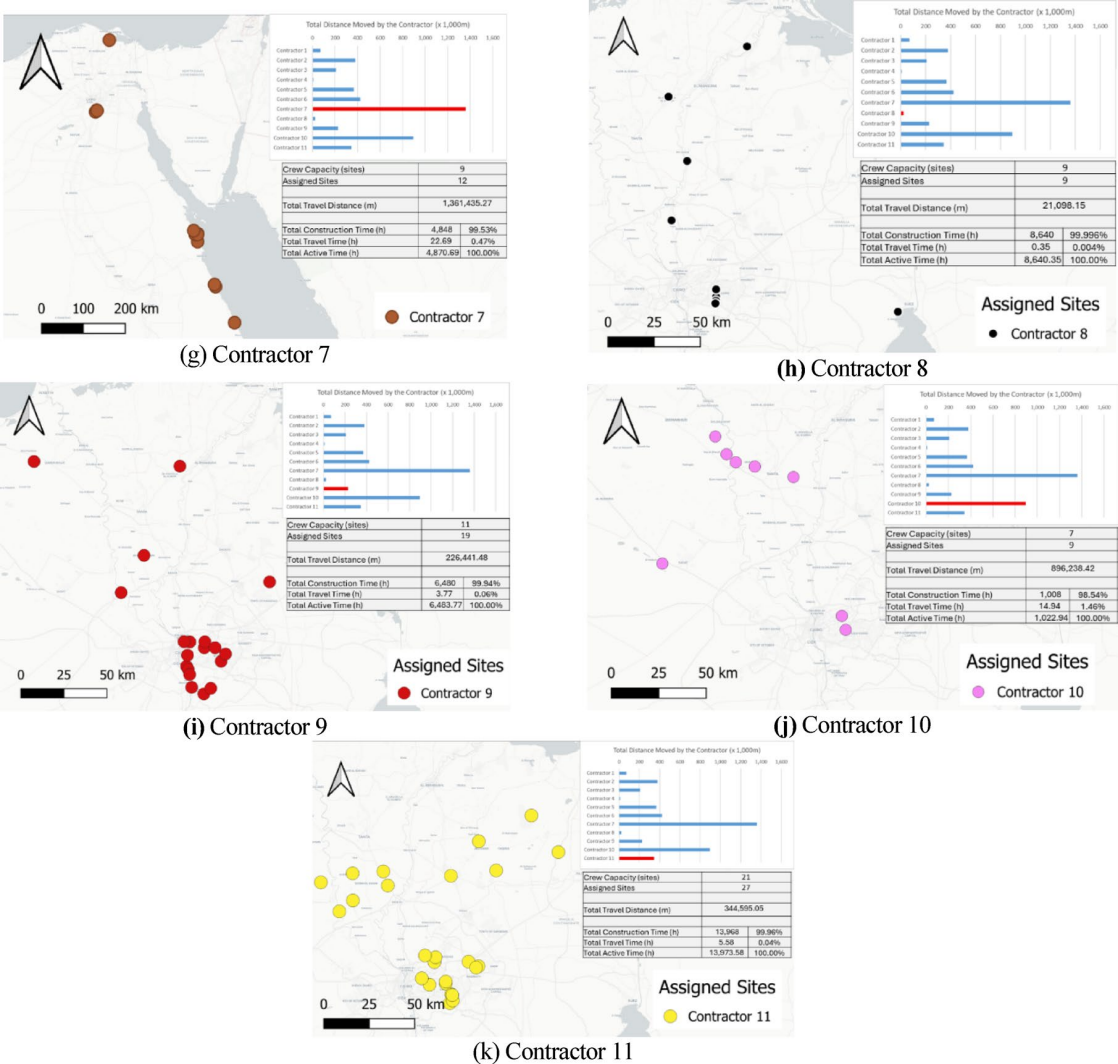
Contractor-specific spatial diagnostics showing assigned site locations with travel-distance and utilization summaries, highlighting clustering and mobility differences. Maps rendered in QGIS 3.36 (LTR) using OpenStreetMap tiles ( © OpenStreetMap contributors, ODbL 1.0; https://www.openstreetmap.org/copyright).

Spatial corroboration was performed using contractor-specific maps (Fig. 14a–k), produced in QGIS 3.36 (LTR) using OpenStreetMap tiles©. These maps visually confirm that most contractors operate within well-defined geographic clusters, while a small number of contractors, particularly 6, 7, 10, and 11, display isolated long-distance assignments corresponding to the analytically flagged far-site cases.

The contractor assignment mechanism is formalized in the following pseudocode to explicitly clarify the decision logic that gives rise to occasional long-distance allocations. The model is structured to minimize total project makespan under contractor specialization and capacity constraints and therefore does not impose explicit penalties or upper bounds on travel distance. When the nearest qualified contractor is occupied, the assignment of any available compatible contractor—even if geographically distant—avoids delaying site initiation and thus prevents prolongation of the overall project duration. Furthermore, because contractors do not return to their depots once mobilized, infrequent long-distance reallocations occurring late in the schedule incur only limited temporal impact on the remaining workflow.

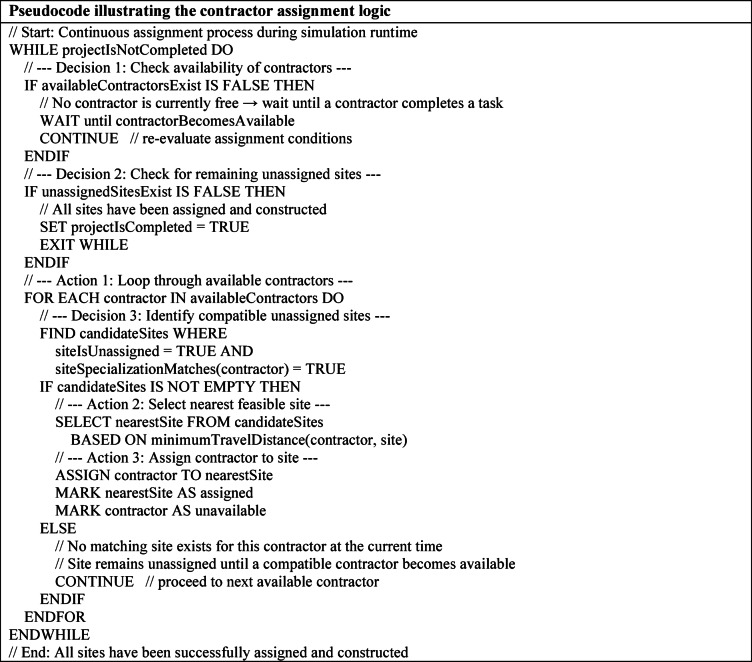



Taken together, these results reinforce that the routing-and-allocation heuristic functions correctly, that deviations are rare and logically justified, and that the model faithfully captures the spatial–temporal trade-offs inherent to SARPs. This spatial validation, combined with the earlier numerical and visual checks, further demonstrates that the GIS-enabled ABM framework—coupled with the embedded GA optimization layer—produces realistic routing behavior and provides meaningful diagnostic insights for geographically dispersed construction programs.

### Model verification

Model verification assessed whether the simulation engine, assignment logic, and data-handling pipelines operate correctly and produce behavior consistent with engineering intuition. A structured set of scenario experiments was executed by modifying contractor capacities and specializations while holding all other parameters constant (e.g., risk settings, travel speed, work calendar). This approach tests whether the model responds logically—such as longer makespans under reduced capacity or stable schedules under balanced specialization—and ensures that routing, state transitions, and risk propagation behave coherently across different crews’ configurations.

#### Scenario design

Five scenarios were tested: the base case and four perturbed configurations that altered total capacity, distribution of crews, and specialization rules (Table 6).

- **Scenario 0 (base case)** – Actual contractor roster: 97 crews across 11 contractors, the reference case (Table 3).
- **Scenario 1 (Sc-1: reduced capacity)** – Contractor and crew counts were significantly reduced, which is expected to increase project duration and increase sensitivity to undercapacity.
- **Scenario 2 (Sc-2: moderate capacity)** – Contractor merger yielding ~ 100 crews, testing whether results interpolate logically between Sc-1 and the base case.
- **Scenario 3 (Sc-3: fixed capacity with specialization)** – Same total crews as base case but each contractor restricted to one site type, probing the model’s handling of specialization constraints.
- **Scenario 4 (Sc-4: expanded capacity)** – Additional contractors/crews to evaluate whether added crews accelerate completion or simply generate more idle time under bottlenecked work scopes.

**Table 6:** Contractor Crew Distribution Across Scenarios.

Scenarios	Contractor	Greenfield	Rooftop	Sharing	Total
Sc-1	Contractor 1	2	0	0	2
	Contractor 2	0	6	2	8
	Contractor 3	1	4	2	7
	Contractor 4	3	0	0	3
	Contractor 5	0	5	2	7
	Contractor 6	7	7	4	18
	Contractor 7	0	0	6	6
	Total crews				51
**Sc-2**	Contractor 1	8	0	4	12
	Contractor 2	12	0	0	12
	Contractor 3	8	5	0	13
	Contractor 4	0	10	0	10
	Contractor 5	0	5	5	10
	Contractor 6	0	0	15	15
	Contractor 7	0	0	12	12
	Contractor 8	4	2	2	8
	Contractor 9	0	8	0	8
	Total crews				100
Sc-3	Contractor 1	7	0	0	7
	Contractor 2	0	8	0	8
	Contractor 3	0	0	6	6
	Contractor 4	0	0	7	7
	Contractor 5	0	7	0	7
	Contractor 6	9	0	0	9
	Contractor 7	9	0	0	9
	Contractor 8	0	0	11	11
	Contractor 9	0	7	0	7
	Contractor 10	21	0	0	21
	Contractor 11	0	0	5	5
	Total Teams				97
Sc-4	Contractor 1	5	1	1	7
	Contractor 2	2	4	2	8
	Contractor 3	2	2	2	6
	Contractor 4	2	2	3	7
	Contractor 5	2	2	3	7
	Contractor 6	3	4	2	9
	Contractor 7	8	1	0	9
	Contractor 8	5	2	4	11
	Contractor 9	1	4	2	7
	Contractor 10	10	8	3	21
	Contractor 11	0	0	5	5
	Contractor 12	6	0	0	6
	Contractor 13	0	6	0	6
	Contractor 14	0	0	6	6
	Total teams				115

Because the framework is data-driven, verification required only replacing the contractor input table; the model reinitialized automatically within seconds. All scenario runs used identical stochastic settings, travel assumptions, and working-time calendars to ensure comparability. Verification focused on three aspects: (i) Monotonicity – Outputs should worsen under reduced capacity and stabilize or improve under balanced expansions, (ii) Logic Conservation – No double assignments, no orphaned sites, and correct state transitions (*Idle → UnderConstruction → Completed*), and (iv) Internal Consistency – Travel and risk delays should aggregate correctly; timers and event triggers must close.

#### Results analysis

**Fig. 15:**
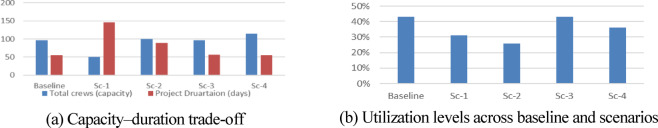
Key scenario outcomes.

Across all five scenarios, the model exhibited coherent and interpretable behavior. The model behaved as expected across all scenarios. Reducing contractor capacity significantly extended project duration (Fig. 15a), while specialization (Sc-3) constrained assignments but still maintained schedules close to baseline—evidence that the allocation logic respects type eligibility. Expanding capacity (Sc-4) did not accelerate completion, confirming that unbalanced crews’ growth does not automatically improve performance. No anomalous behaviors (e.g., orphaned sites, unclosed transitions) were detected.

Key metrics—duration, utilization (Fig. 15b), idle crew-days, and travel hours—shifted consistently with scenario perturbations (Table 7). For instance, fleet utilization dropped under overcapacity (Sc-4) and increased slightly under specialization (Sc-3), while understaffing (Sc-1) inflated idle time despite the smaller workforce. Across all scenarios, travel accounted for less than 0.3% of total active time because construction operations dominate activity duration. No violations of routing logic, specialization eligibility, or continuity constraints were observed. All state transitions closed properly, no site remained unassigned, and travel-distance aggregates matched GIS calculations, confirming the correctness of the underlying event logic.

**Table 7:** Comparative Summary of Scenario Results.

Metric	Base case	Sc-1	Sc-2	Sc-3	Sc-4
Project duration (days)	55	146	89	56	55
Total crews (capacity)	97	51	100	97	115
Busy crew-days	2,269	2,288	2,271	2,284	2,269
Theoretical crew-days (capacity * duration)	5,335	7,446	8,900	5,432	6,325
Fleet utilization	43%	31%	26%	43%	36%
Idle crew-days	3,066	5,158	6,629	3,051	4,056
Total travel hours	71.5	130.8	43.9	41.5	84.2
Average travel share of busy time	0.13%	0.24%	0.08%	0.08%	0.15%
Longest greenfield queue (days)	0.7	11.4	4.3	0.9	1.1

Scenario swap times were minimal: 63.98 (Sc-3) –155.26 (Sc-1) seconds per run, depending on complexity. The baseline scenario completed in 89.1 s, while Sc-2 required 126.95 s, Sc-4 completed in 74.85 s, and Sc-3 achieved the fastest execution at 63.98 s. Even the most demanding scenario (Sc-1) completed in under three minutes on a standard workstation. This performance enables interactive “what-if” exploration during planning meetings without requiring high-performance computing. Overall, the monotonic and diagnostically consistent behavior across scenarios confirms that the model is correctly implemented, logically robust, and suitable for managerial decision support.

### Baseline selection and comparative framework

The Excel-based heuristic schedule used as a benchmark represents a carefully designed and relevant baseline rather than a simplistic or arbitrary method. It is built upon a conventional CPM framework and explicitly incorporates risk-related activities based on expert judgment, capturing uncertainties that are critical to the case studies. In contrast, traditional scheduling tools and CPM-based approaches generally overlook such risk factors. By integrating these considerations, the Excel-based heuristic provides a robust foundation for evaluating the proposed ABM–GA framework, allowing for a rigorous assessment of improvements in performance, resource allocation, and risk-adjusted scheduling outcomes.

## Discussion

The validation results demonstrate that the proposed ABM–GIS simulation–optimization framework achieves substantial makespan reductions and improved operational profiles for SARPs. Applied to a 138-site telecom deployment program, the model reduced the makespan by 46.25% relative to the practitioner’s 100-day Excel CPM-based baseline while using the same contractor pool and field constraints. This improvement is driven by continuity-preserving redeployment and proximity-aware reassignment behavior (nearest-feasible logic), which tends to reduce return-to-base movements and limit idle gaps as emergent effects of the operational decision rules logic —behaviors that traditional heuristic planning fails to exploit.

Across 20 Monte Carlo replications, makespan remained stable within a narrow 49–56-day band, with tight CI and no structural divergence in the critical path. This low variance indicates that the observed improvements are systematic rather than driven by a single-run anomaly, and that the scheduling logic remains robust under stochastic uncertainty. Beyond duration savings, the framework provides diagnostic value. Contractor-level analytics—utilization, travel share, daily state transitions, and spatial assignment maps—reveal operational inefficiencies that are not visible in spreadsheet-based schedules, enabling targeted managerial interventions. Approximately 3% of assignments deviated from strict nearest-contractor proximity; these cases are explainable because the optimization objective prioritizes makespan and does not explicitly penalize long travel when it does not affect the project finish date, suggesting directions for future enhancements (e.g., distance-weighted objectives, travel caps, or multi-objective formulations).

Relative to CPM or spreadsheet-based planning, the framework contributes three integrated capabilities essential for SARPs: (i) Proximity-based, rule-driven assignment that adapts to geographic fragmentation, (ii) GIS-informed travel estimation embedded directly into the simulation, and (iii) Stochastic responsiveness to site-level disruptions.

Academically, this work extends construction simulation research into a domain—scattered, heterogeneous, short-duration repetitive work—that has received limited attention. Practically, it is deployable: all scenarios ran in under two minutes on a standard workstation, enabling rapid “what-if” testing during planning and coordination.

## Limitations and future work

While the proposed framework demonstrates substantial improvements in schedule performance for SARPs, several simplifying assumptions limit its realism and scope. First, the current implementation focuses exclusively on time-related performance; costs, fuel consumption, and emissions are not modeled, preventing integrated time–cost trade-off analysis. Second, contractor heterogeneity is simplified: crews are assumed to have constant productivity and availability, excluding variability arising from experience, equipment readiness, or site-specific constraints. Third, travel-time estimation relies on static numerical distance inputs from the Google Maps Distance Matrix API, without representing congestion, time-of-day effects, or weather-related disruptions that may affect travel in dense urban regions. Fourth, the assignment objective minimizes makespan without explicit penalties for long-distance dispatching, which explains the few far-site allocations observed in the results. Finally, the analysis is based on a single real-world dataset (138-site telecom rollout), limiting external validity until further cases are evaluated.

Future work will address these limitations by:

1. Integrating cost and multi-objective optimization, enabling simultaneous evaluation of time, cost, travel distance, and emissions through dynamic rate tables and Pareto-front analysis.
2. Enhancing assignment logic with travel caps, soft distance penalties, load-balancing rules, and limited look-ahead mechanisms to reduce occasional long-distance deployments.
3. Richer uncertainty modeling, including stochastic productivity, correlated risk events, traffic/weather variability, and systematic robustness experiments.
4. Operational extensions and digital integration, such as variable shifts, overtime, learning/forgetting effects, GPS-based tracking, and digital-twin pipelines for continuous re-optimization.
5. Broader benchmarking to establish a more comprehensive performance baseline and strengthen generalizability.

Overall, while the framework already provides a practical, auditable tool for planning scattered short-duration typical/atypical repetitive projects, these extensions will deepen analytical fidelity and broaden its applicability across diverse project environments.

## Conclusions

This paper presented an ABS-GA optimization framework for planning and scheduling SARPs. The model integrates GIS-enabled routing, contractor-specific specialization, stochastic disruptions, and spatially guided crew assignments to represent field behavior with greater realism. Applied to a real 138-site telecom rollout across Cairo, the Delta, and Sinai, the framework consistently reduced the project makespan from 100 days to approximately 53.75 days (46.25% improvement) under identical constraints. These gains were driven by continuity-preserving dispatching and adaptive nearest-site reassignment, which tended to reduce idle gaps and avoid return-to-base movements as emergent effects of the redeployment logic.

The framework’s value extends beyond duration reduction. It provides interpretable diagnostics—utilization profiles, travel-effort distributions, spatial assignment patterns—and executes full scenarios in under two minutes on a standard workstation, enabling practical, iterative “what-if” analysis for and planners. Only a small share of assignments deviated from strict geographic proximity, an expected outcome given that the optimization objective targets makespan rather than travel distance; these cases transparently highlight opportunities for future distance-aware extensions. Methodologically, the study contributes a deployable simulation–optimization approach tailored to multi-contractor, spatially fragmented projects—an area where traditional CPM/LOB or deterministic heuristics struggle to capture routing, specialization, and uncertainty in an integrated manner.

Limitations include simplified travel-time estimation, the absence of explicit cost modeling, and validation based on a sole case study. Future work should incorporate multi-objective time–cost–distance trade-offs, richer uncertainty representations, and expanded benchmarking performance against alternative scheduling/optimization approaches.

Overall, the results indicate that ABS coupled with optimization can provide actionable decision support for geographically dispersed repetitive programs by producing feasible schedules under uncertainty and enabling replication-based comparison with practitioner baselines, thereby improving upon conventional manual scheduling and supporting more advanced, data-driven decision construction logistics planning.

## Supplementary Information

Below is the link to the electronic supplementary material.

Supplementary Material 1

Supplementary Material 2

Supplementary Material 3

Supplementary Material 4

## Data Availability

Sample input datasets (siteData.csv and contractorsData.csv), the full pseudocode for Algorithms 1 and 2, and the complete 20-run Monte Carlo replication results are provided as Supplementary Files accompanying this manuscript. These materials enable full methodological replication of the reported experiments. All procedural steps required for independent re-implementation of the proposed framework are described in detail within the manuscript and the supplementary documentation. Additional clarification or supporting materials may be made available from the corresponding author upon reasonable request.

## Abbreviations

SRPs: Scattered Repetitive Projects
SARPs: Scattered Atypical Repetitive Projects
CPM: Critical Path Method
LOB: Line of Balance
LSM: Linear Scheduling Method
GIS: Geographic Information System
ABM: Agent-Based Modeling
GA: Genetic Algorithms
MRCTCTP-RP: Multi-Mode Resource-Constrained Time–Cost Tradeoff Problem for Repetitive Projects
MTSP: Multiple Traveling Salesman Problem
PSO: Particle Swarm Optimization
ABS: Agent-Based Simulation
FSM: Finite State Machine
KPIs: Key Performance Indicators
CI: Confidence Intervals
GF: Greenfield
RT: Rooftop
*n*: Number of scattered sites
*x*: Number of site types
*P*: Probability of a stochastic risk event
δ: Impact of a stochastic delay (in days)
$$\bar{x}$$: Mean value
*S*: Standard deviation
μ: Mean distance
