# Supplementary material for: Agent-based simulation for multi-resource-constrained scheduling of scattered atypical repetitive projects
Source: Sci Rep. 2026 Apr 3;16:11759. doi: 10.1038/s41598-026-42832-1 (PMC13066151; doi:10.1038/s41598-026-42832-1)
Supplement: Supplementary file 4 — Supplementary Material 4 [file 41598_2026_42832_MOESM4_ESM.pdf]

Supplementary File S1 – Supplementary Algorithms

Manuscript ID: e8ebbde0-9007-472a-86e9-c93dbddf4f66

Title: Agent-based simulation for multi-resource-constrained scheduling of scattered atypical repetitive projects

Authors: Rawan Abdelrahman Sultan, Khaled Hamdy, Yasmeen A. S. Essawy

This Supplementary Information file reports the pseudocode for the two main routines governing site progress in the model: Algorithm S1 (*Idle → UnderConstruction*) and Algorithm S2 (*UnderConstruction* → Completed), including the key inputs, guard conditions, and outputs recorded for analysis.

A. Supplementary Algorithm 1 (*Idle → UnderConstruction* transition)

As formalized in Algorithm 1, the site execution routine (i) computes travel distance/time from the contractor's current location using the selected distance method and working-time assumptions, (ii) applies stochastic disruption delays based on the specified risk parameters, and (iii) accounts for non-working days to convert effort into elapsed simulation time while logging key schedule events. This routine ensures that each site's completion time reflects both spatial mobility and uncertainty in a parameter-controlled manner.

Algorithm 1 (*from Idle to UnderConstruction)*

```
Inputs / Global Parameters (set by user or experiment design)
    PARAM SPEED_KMPH
    PARAM HOURS_PER_DAY
    PARAM WORKDAYS_PER_WEEK
    PARAM HOLIDAYS_SET
    PARAM RISK_PROB
    PARAM RISK_DELAY_DIST
    PARAM DISTANCE_METHOD

PROCEDURE StartConstruction(siteID)

    Log Construction Start
    PRINT "Construction started for site", siteID

    Initialize construction start time (only once at beginning)
    CURRENT_SIM_TIME() → constructionStartTime

    Calculate distance between contractor and site (in KM)
    CEIL( Distance(assignedResource, site) / 1000 ) → distance

    Calculate travel time in days (assumed 60 km/h speed, 8 hours/day)
    CEIL( (distance / 60) / 8 ) → travelTime
    PRINT "Travel Time:", travelTime, "days for site", siteID
```

Simulate stochastic risk event with 20% probability, assuming delay 1-10 days
IF RANDOM_PROBABILITY(0.2) = TRUE THEN
    UNIFORM_INT(1, 10) → riskDelay
    PRINT "Risk event occurred at site", siteID, "Delay:", riskDelay, "days"
ELSE
    0 → riskDelay
END IF

Calculate paused days
((CURRENT_SIM_TIME() MOD 7) + 1) → weekDayOffset
FLOOR((travelTime + constructionDuration + riskDelay + weekDayOffset - 2) / 6) → pausedDays

END PROCEDURE

B. Supplementary Algorithm 2 (*UnderConstruction → Completed*)

The transition formalized below in Algorithm S2: it (i) finalizes completion by marking the site as completed, (ii) computes and records the realized completion time by aggregating planned duration with travel time, stochastic disruption delays, and non-working-day pauses, and (iii) logs site-level delay components and updates contractor performance counters by site type before releasing the contractor back to the assignment pool.

Algorithm 2 *(from UnderConstruction to Completed)*

PROCEDURE CompleteConstruction(siteID)

Mark site as completed
isCompleted → TRUE

Log completion and total delay
PRINT "Construction completed for site: ", siteID, "by contractor:", assignedResource
PRINT "Total delay for site ", siteID, ": ", (riskDelay + pausedDays), " days"

Compute final construction time (start + planned + risk + pauses + travel)
totalConstructionTime → constructionStartTime + duration + riskDelay + pausedDays + travelTime

Log and store final construction time
PRINT "Final construction time for site:", siteID, "is", totalConstructionTime, "days"
this.totalConstructionTime → totalConstructionTime

Log contractor wrap-up
PRINT "Contractor", assignedResource.contractorName, "finished site:", siteID

Increment contractor counters by site type

```
    IF siteType = "Sharing" THEN
        assignedResource.sharing → assignedResource.sharing + 1
        ELSE IF siteType = "GreenField" THEN
            assignedResource.greenfield → assignedResource.greenfield + 1
        ELSE IF siteType = "Rooftop" THEN
            assignedResource.rooftop → assignedResource.rooftop + 1
        ELSE
            NULL
    END IF

    Release contractor
    assignedResource → NULL

END PROCEDURE
```
